# Statistical Study of the Performance of Recursive Bayesian Filters with Abnormal Observations from Range Sensors

**DOI:** 10.3390/s20154159

**Published:** 2020-07-26

**Authors:** Manuel Castellano-Quero, Juan-Antonio Fernández-Madrigal, Alfonso-José García-Cerezo

**Affiliations:** Systems Engineering and Automation Department, University of Málaga, 29071 Málaga, Spain; jafernandez@uma.es (J.-A.F.-M.); ajgarcia@uma.es (A.-J.G.-C.)

**Keywords:** Bayesian filters, range sensors, abnormal observations, mobile robots

## Abstract

Range sensors are currently present in countless applications related to perception of the environment. In mobile robots, these devices constitute a key part of the sensory apparatus and enable essential operations, that are often addressed by applying methods grounded on probabilistic frameworks such as Bayesian filters. Unfortunately, modern mobile robots have to navigate within challenging environments from the perspective of their sensory devices, getting abnormal observations (e.g., biased, missing, etc.) that may compromise these operations. Although there exist previous contributions that either address filtering performance or identification of abnormal sensory observations, they do not provide a complete treatment of both problems at once. In this work we present a statistical approach that allows us to study and quantify the impact of abnormal observations from range sensors on the performance of Bayesian filters. For that, we formulate the estimation problem from a generic perspective (abstracting from concrete implementations), analyse the main limitations of common robotics range sensors, and define the factors that potentially affect the filtering performance. Rigorous statistical methods are then applied to a set of simulated experiments devised to reproduce a diversity of situations. The obtained results, which we also validate in a real environment, provide novel and relevant conclusions on the effect of abnormal range observations in these filters.

## 1. Introduction

Range sensors are nowadays present in numerous tasks involving perception of the environment. These devices are employed within a wide variety of applications related to industrial manufacturing [1], autonomous driving [2] and robotics [3], among many other fields. Regarding the latter, rangefinders play a role of capital importance as part of the sensory apparatus of many robots used in industrial, rescue and service tasks [4,5,6]. In particular, mobile robots usually operate in complex scenarios where they are required to navigate safely and autonomously. A proper sensory perception is essential, however, it is often challenging for a range sensor to work under real conditions, due to the uncertain nature of the environment to be captured and the limitations of the sensor itself.

One of the most well-known shortcomings has to do with the impossibility of getting an exact value of any distance, since all the measurable quantities of the physical world are subjected to some degree of unpredictability. This issue has been extensively treated and it is traditionally addressed by applying *estimation* theory [3]. There exist numerous kinds of estimators depending on the nature of the stochastic process to be considered (please refer to Reference [3] for a more in-depth treatment). In the case of mobile robotics, it is common that the variables that need to be estimated evolve over time, such as the distances measured by a range sensor. The most common dynamic estimators used in robotics are based on Bayesian probability theory, particularly on Recursive Bayesian Estimation (RBE). Among the inference tasks that Bayesian estimation can handle, *filtering* is particularly common in robotics. The concrete methods used for that are employed to solve many different problems, such as localization, navigation and mapping. They are considered essential for a robot to work properly, and the quality of sensory observations is therefore critical for them.

Unfortunately, the impossibility of measuring actual, exact and deterministic distances is not the only issue affecting sensory data from rangefinders. As mentioned before, mobile robots operate in real world scenarios, where they are exposed to a wide variety of situations that might lead to corrupt sensory data in not fully stochastic ways. These *abnormal* effects, in contrast to *noisy* ones, are often provoked by intrinsic limitations of the sensory apparatus, and related to the measurement principles of physical devices. For instance, a sensor relying on the detection of infrared radiation will not be able to perceive obstacles with transparent or absorbent surfaces, nor operate nominally in conditions of extreme sunlight, leading to saturated or missing observations. Challenging parts of a scene such as corners or columns could also affect ultrasonic sensors, for example, by altering the way that their emitted mechanical waves are reflected, leading to measured distances larger than the actual ones, that is, biasing them.

Our aim in this work is to study and quantify the impact of common abnormal range observations on the performance of Bayesian filters. There exist already some works in the literature that partially cover the study of filtering performance, such as References [7,8,9], where the convergence and accuracy of some Bayesian estimators is addressed. From an analytical point of view, these works provide sufficient conditions related to the estimation error and innovation in order to ensure convergence; however, they do not take into account the presence of the abnormal effects we considered here, that may potentially modify or invalidate the established conditions. They also restrict to the case of a particular filter and do not study any further aspect of the performance. There exist some other works in the literature that address the case of anomalous observations by developing strategies to identify and recover from them, such as References [10,11,12]; however, these contributions lack a complete analysis of the impact of such sensory data on different aspects of the filtering performance.

To address the mentioned issues, we contribute in this work with a thorough statistical study that analyses and quantifies the effects of common abnormal observations from range sensors on the performance of Bayesian filters. Since our aim is to cover a broad variety of filtering models, we address the estimation problem from a generic perspective that allows us to abstract from the concrete implementation of any estimator, such as Kalman or Particle filters [13,14,15]. For that, we use the rigorous probabilistic framework provided by dynamic Bayesian networks [16], which is capable of representing arbitrary causal relationships among random variables and enables for generic inference, that is, they can play the role of any Recursive Bayesian Filter (RBF).

The reason an analytical approach is not advisable for this problem is that a large number of parameters have to be considered in order to study a sufficient variety of abnormal situations (e.g., the conditions of the filtering problem, the sensor modelling parameters, the amount and value of anomalous sensory data, etc.). An analytical derivation would be cumbersome under these conditions, and possibly impractical. To solve this issue, from an alternative, statistical approach, we first analyse the most common abnormal situations that affect range sensors, define several parameters that serve to assess the performance of the filters, and also define the factors (anomalies and system parameters) that are likely to modify such performance. Then we use rigorous statistical methodologies applied to sets of simulated experiments designed to reproduce a wide variety of situations. The obtained results provide us with complete and relevant conclusions about the effects of dealing with sensory abnormal observations, in a flexible way and without loss of generality. In this paper, we also validate the obtained conclusions in a real scenario with a mobile robot.

The rest of the paper is organized as follows. Section 2 reviews some works related to Bayesian frameworks for estimation, sensory abnormal behaviour and performance of filters. Section 3 sets the theoretical background related to the estimation problem, introduces the statistical methodologies used in this work and describes the procedure we have followed to obtain simulated data. Section 4 provides a complete statistical analysis and experimental validation of the results of our study, both in simulation and in reality. Finally, Section 5 summarizes the main contributions of the paper and proposes future work.

## 2. Related Works

The study of the intrinsic limitations and external abnormal conditions that may affect exteroceptive range sensors has been extensively treated in the literature. However, most of the existing references do not address this issue in isolation; instead, they provide a broader insight ranging from the very physical principles of measurement to concrete applications. One of the first and most complete reviews on sensing technologies in mobile robotics can be found in Reference [17]. More recently, complete classifications of these sensors according to their applications appear in texts such as Reference [18]. Considering the wide variety of existing exteroceptive rangefinders, these classifications could be roughly divided according to two mains aspects, namely, the number of spatial dimensions the sensor is able to deal with and the nature of waves it uses (e.g., ultrasonic, electromagnetic, etc.). Measurement principles of most single-direction rangefinders are reported in Reference [3] along with the main limitations they may suffer. Regarding higher dimensional rangefinders, physical working principles are addressed in Reference [19], where the most common abnormal observations they may yield and their causes are also covered, as well as in Reference [3]. Another important aspect to our work concerning sensor modelling is its characterization from a probabilistic perspective, which is tackled in Reference [18] and Reference [3].

Bayesian estimation is a powerful tool for dealing with the noisy nature of the data gathered by these sensors. In general, it can be found within a wide variety of applications in different disciplines such as economics [20], biomedicine [21], physics [22] and engineering [23], among others. In this work we are particularly concerned about its applications in mobile robotics, whose problems have been identified and deeply treated in the literature [24]. The essential tasks that a robot must perform to work properly and autonomously have been addressed successfully in practice from the incorporation of probability theory to robotics in the late 1990s and early 2000s. It is the case of localization [25], navigation [26] and simultaneous localization and mapping (SLAM) [27]. In order to estimate the *pose* (posture) of a robot while navigating within an unknown environment and building a representation of it at the same time, the use of some kind of proprioceptive or exteroceptive sensors is mandatory; exteroceptive range sensors play a role of capital importance in these problems [3].

Under the global denomination of Recursive Bayesian Estimation (RBE), there exist an important variety of concrete implementations of Bayesian estimation depending on the nature of the stochastic process itself and the assumptions made about it. These implementations are usually classified into two broad groups, namely, *parametric* and *non-parametric* filters, depending on whether a known distribution shape for the uncertainties is assumed or not. Developed in the 1960s, the well-known *Kalman Filter* (KF) [13] was the first contribution to the group of parametric filters. Its assumptions consist mainly on the normality of all uncertainties involved and the linearity of the models it represents. As estimator of the state of dynamic systems, it is also referred to as *linear dynamical system* or *state space model* in the literature [28,29]. Later on, parametric filters allowing the representation of non-linear systems were developed, such as the *Extended Kalman Filter* (EKF) [30], which linearizes such non-linear models while maintaining the assumption of Gaussianity, or the *Unscented Kalman Filter* (UKF) [31], which improves the accuracy of the EKF approximating the original distributions in the non-linear models by using a sampling technique called the *Unscented Transform* [3].

The main limitation of parametric filters relies on the fact that they cannot handle, for instance, uncertainties with multimodal distributions (a mobile robot that estimates that it can be with high probability in one out of several places), and more generally, with non-Gaussian distributions. However, there also have been relevant developments in the scope of non-parametric filters that allow to deal with arbitrary shapes of uncertainty. One of them is the *Histogram Filter* (HF) [25], which is grounded on discrete Bayesian estimation and enables to approximate continuous state spaces (e.g., the so-called *Markov Localization* in mobile robotics). The main drawback is its computational cost, which is usually solved by considering the use of one solution belonging to the family of the *Particle Filters* (PF), the most relevant development in this scope (e.g., *Monte Carlo Localization*). This denomination stands for all those algorithms relying on Monte Carlo simulation methods, which aim to approximate arbitrary distributions by using random observations from them. One of the first concrete implementations of the PF was called *Sequential Importance Sampling* (SIS), which was refined later on with the introduction of *Sequential Importance Resampling* (SIR) [14]. An important drawback of these sampling-based algorithms is their still high computational cost when the dimensionality of the problem is high, which was alleviated by the development of *Rao-Blackwellised Particle Filtering* (RBPF) [15].

Another area of research related to the study of Bayesian frameworks was producing novel results that would have an important impact on recursive estimation. Developed in the 1980s, *Bayesian Networks* (BNs) [32] are a kind of probabilistic graphical model that allows to compactly represent a joint distribution while encoding independence assumptions. The main implication is, therefore, the possibility of representing arbitrarily complex relationships among random variables, viewed in this context as cause-effect implications, in a flexible and rigorous manner. Numerous inference algorithms (both exact and approximate) were devised for these models, being one of the most relevant ones the exact *junction tree* or *clique tree* algorithm [33]. However, these models were first conceived for discrete variables and static systems only. The introduction of *Dynamic Bayesian Networks* (DBNs) [16] aimed at incorporating the temporal dimension to such a generic representation tool. This notion along with the inference algorithm developed in Reference [34], which extended the inference capabilities to hybrid models (with both discrete and continuous variables), formed the basis for the connection between inference in generalist models and filtering for dynamic systems. This was finally achieved in some relevant works as the one in Reference [28], which contributed with a novel exact inference method for generic DBNs called the *interface* algorithm, based on the traditional *join tree*. That work shows the relations existing between DBNs and Kalman Filters, which are a particular case of the former, and also provides approximate inference algorithms for generic DBNs based on particle filtering.

As mentioned before, our main aim consists in studying the impact of range sensory limitations and abnormalities on the performance of Bayesian filters. There exist related works in the literature that address particular aspects of this issue. On the one hand, some works pursue the identification of abnormal observations and develop solutions to recover from them. This can be seen in Reference [35], where generic anomalous observations are detected and treated for parametric filters. Regarding more specific abnormalities, papers such as Reference [36] and Reference [37] develop robust estimators in the presence of data outliers for parametric and non-parametric filters, respectively. Another common kinds of problematic observations being treated in the literature are the intermittent [10] and biased ones [11]. In previous works [38] we also contributed with a solution based on Bayesian networks that is able to identify and overcome different kinds of sensory anomalies. On the other hand, and from a more theoretical perspective, there exist analytical approaches that study the optimality, sensitivity and performance of filters in case of modelling errors, such as References [39,40], while others address their stability and convergence [7,8], but these works only analyse partial aspects of the filtering performance, such as convergence, doing it without taking into account the effect of possible abnormal observations and also restricting to particular implementations. In this work we aim to cover a broader variety of filtering models while providing a deeper analysis of the performance at the same time, using rigorous statistical methods for that.

## 3. Design and Methodology of the Study

In this section we develop the two main aspects of our study. On the one hand, in Section 3.1 we state the problem to which the generic Bayesian filter will be applied (filtering range sensor observations), along with the necessary theoretical background. We address the filter parameterization, taking into account the modelling of range sensors (whose limitations are covered in Section 3.2), present mechanisms for inference, and introduce the variables that define the filter performance and the factors that might have some influence on it (Section 3.3). All of this constitutes the theoretical aspects to the design of our study. On the other hand, in Section 3.4 we present the statistical methodologies that will be used to derive meaningful and complete conclusions from the study. Also, we describe the procedure to be followed to perform the study as well as the gathering of performance data (Section 3.5 and Section 3.6).

### 3.1. Generic Bayesian Networks for Filtering Range Sensors

Formally, a Bayesian Network (BN) defined on a set of random variables $Z=\{Z_{1},Z_{2},\ldots ,Z_{n}\}$ is a pair (G,Θ) consisting of a direct acyclic graph G over Z, called the *network structure* and a set of Conditional Probability Distributions (CPDs) Θ for each variable in Z, called the *network parameterization*. The graph structure captures the causal relationships existing among variables through directed arcs, which indicate dependencies, and the CPDs define the distributions for the dependent variables. This model compactly encodes the joint probability distribution over Z, which enables us to infer new knowledge from existing one, that is, to deduct P(Q|E), where Q is the set of query variables (variables of interest) and E is the set of observed variables (the existing knowledge), also called the evidence. In this context, Z=Q∪E and Q∩E=∅. For a more in-depth treatment of Bayesian networks please refer to Reference [41].

The previous definition is restricted to static models, but it can be extended to cope with discrete-time stochastic dynamic processes [42]. For that, the timeline is discretized into a set of regularly spaced intervals called *time slices* [29], which represent variables of the system state at different times, and referred to with integer numbers. A 2-time-slice Bayesian network (2-TBN) [42] is a process whose state variables at a certain time *t* are $X^{\left(t\right)}=\left\{X_{1}^{\left(t\right)},X_{2}^{\left(t\right)},...,X_{n}^{\left(t\right)}\right\}$. It is a fragment of a Bayesian network whose structure is defined over the union of state variables at adjacent time slices, that is, $X^{\left(t-1\right)}\cup X^{\left(t\right)}$, and it is only parameterized for those nodes in the graph corresponding to variables $X^{\left(t\right)}$ (thus, only those nodes are annotated with CPDs). Also, nodes referring to variables $X^{\left(t-1\right)}$ have no parents. Actually, this network represents a conditional distribution of the form $P(X^{\left(t\right)}|X^{\left(t-1\right)})$, usually called *transition model*.

A Dynamic Bayesian Network (DBN) [29] can be defined as a pair ($B_{0}$, $B_{\to }$) where $B_{0}$ is a Bayesian network over variables $X^{\left(0\right)}$, which represent the initial distribution of state variables, and $B_{\to }$ is a 2-TBN for the process, also referred to as *transition network*. Note that, given a time span T≥0, this representation allows us to compose the initial network $B_{0}$ along with instances of the transition network $B_{\to }$ to create an equivalent monolithic Bayesian network over all the variables within such time span. This operation is called *unrolling* of the DBN and it is related to some inference methods, which will be discussed later on. For a more in-depth treatment of DBNs and inference please refer to References [28,29,42].

**Figure 1:**
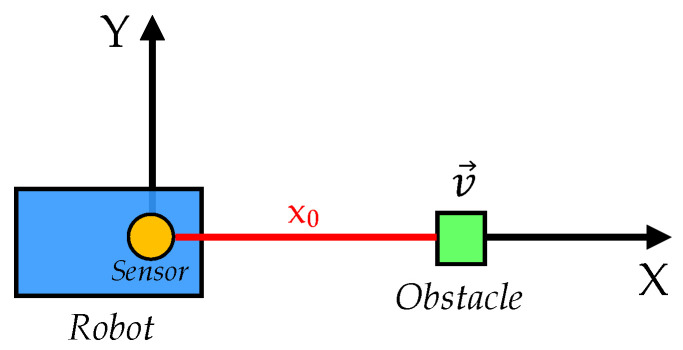
General obstacle tracking problem addressed in this work. Here, $x_{0}$ represents the initial distance to the obstacle, which moves at a constant speed $\vec{v}$ in the positive sense of the *X* axis.

Our aim is to assess the impact of abnormal observations from range sensors on the performance of Bayesian filters. For that, we have to consider a problem where rangefinder sensors are used by Bayesian estimators to access the hidden true distance to some, possibly moving object. This is a common problem in mobile robotics, where the sensor is mounted on-board the robot. Notice that this setting can also fit with many non-robotic applications that use rangefinders. For the sake of simplicity we are only considering one-dimensional movement of the obstacle (along the *X* axis in this case), since this suffices to cover the common abnormal observations that can occur with a rangefinder. In Figure 1 we can see the conditions of the problem, where $x_{0}$ is the initial distance to the obstacle, which moves at a constant speed *v* in the positive sense of the *X* axis.

**Figure 2:**
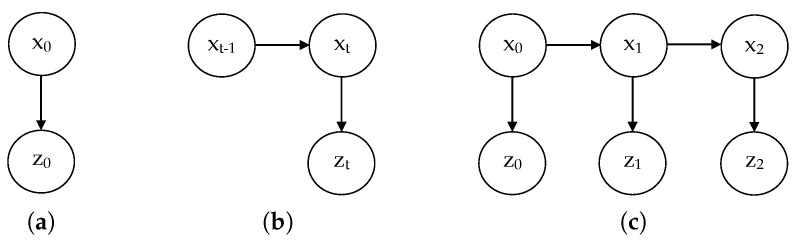
Dynamic Bayesian network corresponding to the obstacle tracking problem. Here, variables *x* represent hidden states (true distances) while variables *z* represent observations (sensor readings). (**a**) Initial network $B_{0}$. (**b**) Transition network $B_{\to }$.(**c**) Unrolled DBN for three time slices.

This problem can be solved by using one of the Bayesian estimators reported in Section 2, such as the Kalman filter. As we have explained, we pursue a more generalist approach and, for this reason, we are constructing an equivalent estimator in the form of a dynamic Bayesian network. For that, we need to consider two different kinds of variables, namely, the ground-truth distance to the obstacle, which is inaccessible (*hidden* variable) and the distance measured by the range sensor, called the *observation*. These variables will be denoted as $x_{t}$ and $z_{t}$, respectively, for a certain time slice *t*. Since the physical quantities involved in this problem are continuous, the variables used will also be continuous random variables. The model structure corresponds, in this case, to the classical one used in Bayesian estimation for continuous variables, called linear dynamical system (LDS) [29], whose representation in the form of a DBN is depicted in Figure 2.

Once the network structure is defined, we have to proceed with its parameterization. In this case, all the variables are continuous and we also assume that the corresponding CPDs are linear-Gaussian, according to the following definition [29]. Let *y* be a continuous random variable appearing as a node in a network with continuous parents $u=\{u_{1},u_{2},\ldots ,u_{n}\}$. A linear-Gaussian CPD for the corresponding node of variable *y* is the distribution:

(1) $$p\left(y|u\right)=N\left(\beta_{0}+\beta_{1}u_{1}+...+\beta_{n}u_{n},\sigma^{2}\right),$$

where $\beta_{0},\ldots ,\beta_{n}\in R$ and N denotes a normal distribution whose mean is a linear combination of parents in u and its variance is $\sigma^{2}$.

The LDS model in Figure 2 needs three different kinds of CPDs. Firstly, we define the one for all nodes *z*, called the *observation* model, that is, $p(z_{t}|x_{t})$. This CPD encodes the probability distribution of the sensory observation given the true position of the obstacle. In other words, what this CPD represents is the noisy behaviour of the range sensor, which depends on the particular device we use. Such behaviour is often modelled with a truncated normal distribution with the same mean as the true position and some standard deviation depending on the particular sensor, given by the manufacturer in terms of accuracy, which is the error between the measured distance and the actual one. In this work we aim to cover as much actual sensors as possible. We compile in Table 1 a representative list of commercial rangefinders commonly used in mobile robotics [3].

Based on the accuracy reported for each sensor (Table 1), we use in our simulation framework its average in order to represent most of them. Therefore, the standard deviation for our observation model is σ≈6 cm, that is, we are considering that approximately 68% of the measures will have that error at most. Also, we have chosen such value because 2σ≈12 cm, which is the worst accuracy in Table 1. This way, all the representative sensors in the table are covered, meaning that 95% of the measures will have that worst error at most. The CPD of the observation model for a given time slice *t* is:

(2) $$p\left(z_{t}|x_{t}\right)=N\left(x_{t},\sigma^{2}\right),$$

where σ=0.06 m (we will always parameterize the CPDs in SI units).

Now we focus our attention on the corresponding CPD for the transition model, that is, p$\left(x_{t}|x_{t-1}\right)$. Considering our obstacle tracking problem (Figure 1), and the lack of any further proprioceptive information on the robot motion (we are not interested in this study on the filters ability to fuse information), the actual distance to the obstacle at a certain time slice *t* can be expressed in terms of the previous one t−1 with a simple “constant velocity” model:

(3) $$x_{t}=x_{t-1}+v\Delta t,$$

where *v* is the constant speed of the obstacle and Δt is the time span between subsequent slices, also constant. Thus, the CPD for the transition model becomes:

(4) $$p\left(x_{t}|x_{t-1}\right)=N\left(x_{t-1}+v\Delta t,\epsilon \right),$$

where ϵ is small because we assume a highly accurate proprioceptive measurement of the speed *v* in this model [3].

At this point we only lack the prior distribution for the initial state variable $x_{0}$ (Figure 2). We assume that the actual initial position of the obstacle is unknown, thus, the corresponding CPD must be a normal distribution with a high variance, at least much greater than the variance of the observation model, close to a uniform distribution. We have opted for the average central point of the measurement range for sensors in Table 1 as the mean, which is 2 m approximately, and a standard deviation 200 times greater than the one for the observations (approximately equal to 12 m). The resulting CPD is:

(5) $$p\left(x_{0}\right)=N\left(2,12^{2}\right).$$

Once the parameterization of our model is complete, it is possible to perform inference. In the context of dynamic Bayesian networks, there exist different kinds of queries that can be formulated for an inference task (see Reference [28] for a complete review). However, in this work we are only interested in the one of filtering. This query consists in calculating the posterior distribution of the current actual position given the whole history of observations, from the initial state up to the present. Such distribution is of the form p$\left(x_{t}|z_{0:t}\right)$, where $z_{0:t}=\left\{z_{0},z_{1},...,z_{t}\right\}$. Since our aim is the study of generic filters, we use an inference method called the *interface algorithm* [28], which is is able to deal with arbitrary architectures of dynamic Bayesian models.

### 3.2. Sensory Anomalies and Limitations

According to the literature on range sensors presented in Section 2, the most common signs of anomalous sensory behaviour appear mainly in the form of biased observations and saturated or missing ones. The first kind is common, for instance, in some ultrasonic rangefinders when placed next to corners or similar surfaces. These sensors rely on the reception of some previously emitted mechanical wave, which would reflect too many times under abnormal conditions before reaching the receptor, thus leading to a detected distance larger than the actual one.

However, this issue does not only affect ultrasonic range sensors, but also the ones relying on infrared radiation. There exist common situations in real environments where mobile robots are placed nearby transparent or highly specular surfaces. As in the case of ultrasonic sensors, these kind of devices usually wait for the reception of a previously emitted pulse of light. This radiation is not sensitive to the case of transparent surfaces, such as windows, therefore ignoring their presence and possibly leading to a larger distance depending on the particular scene behind. Similarly, specular surfaces would deviate this pulse of light towards nearby obstacles, leading again to larger distances than the actual ones depending on the concrete features of the scenario.

The second abnormal issue, that is, the presence of missing observations, is also common in both ultrasonic and light-based sensors. Under undesirable circumstances, the emitted wave (either mechanical or electromagnetic) could be absorbed or reflected by specific kinds of surfaces in such a way that the receptor is not reached, thus provoking a false indication of free space. There also exist another issue concerning sensors relying on infrared light, related to the presence of external sources of the same radiation. For instance, in conditions of extreme sunlight or heat, the wave emitted by the device would suffer from interferences with the natural radiation, leading again to false indications of free space.

### 3.3. Filter Performance Measures and Problem Characterization

According to Reference [3], there are some important aspects regarding the performance of any kind of Bayesian estimator, namely, how good it is as an approximation to the value of interest, how much uncertainty it has, and how we expect it converges to the actual value as more and more observations are gathered. We now quantify each of these aspects.

The first one can be defined as the accuracy of the filter, that is, the error between the predicted value and the actual one. More formally, let $\mu_{t}$ be the actual distance to the obstacle being tracked at time *t*, and let ${\hat{\mu }}_{t}$ be the estimated distance, which corresponds to the mean of the normal distribution represented by the posterior p$\left(x_{t}|z_{0:t}\right)$. The accuracy of the filter $e_{t}$ at a given time slice *t* (also called *step* within this scope) is then:

(6) $$e_{t}={\hat{\mu }}_{t}-\mu_{t}.$$

Note that the value $\mu_{t}$ is non-observable in reality. We can handle it in this work thanks to the nature of our simulated statistical study (see Section 4 for further implementation details).

The second aspect to the performance of a Bayesian estimator is its uncertainty, which in this case takes the value of the standard deviation of the normal distribution represented by p$\left(x_{t}|z_{0:t}\right)$. We will denote it as $\sigma_{t}$, for a given time step *t*.

The last aspect we consider is related to the convergence of estimations to the actual value. Defining a measure that represents convergence is not as straightforward as in the previous cases, and there are several solutions we could adopt. The term convergence usually refers to the minimum number of steps to be taken in the filtering process such that some desirable behaviour is reached. We characterize such behaviour inspired by the time response of dynamical systems [49]. Particularly, we consider that a Bayesian estimator converges for a number of steps $t^{*}$ if the absolute value of the difference between adjacent errors $|e_{t^{*}}-e_{t^{*}-1}|$ becomes smaller than a specified threshold and if this still holds for the remaining steps $t\geq t^{*}$ (note that we need the full sequence of observations to check that, thus, we must do it offline). The concrete implementation of this measurement as well as the calculation of a proper threshold will be addressed in Section 4.

The accuracy and uncertainty have been defined so far as a function of the concrete time step *t*; however, our aim is to characterize such performance by using only one value that represents the overall quality of the resulting estimation. For that, we define the *expected* accuracy and uncertainty of a filter ($\bar{e}$ and $\bar{\sigma }$, respectively) as the values of accuracy and uncertainty that are expected to be achieved when the filtering process has converged (there is no point in considering the case of divergent estimations, since the mentioned values would increase indefinitely). We will estimate them by taking the mean values of accuracy $e_{t}$ and uncertainty $\sigma_{t}$ achieved for the last 10% of time steps in the filtering process.

As a summary, the three measures of performance we have defined are:

- Expected accuracy of the filter ($\bar{e}$).
- Expected uncertainty of the filter ($\bar{\sigma }$).
- Minimum number of steps that lead to convergence ($t^{*}$).

In this work we need to define a set of factors that might potentially affect these measures of performance. Regarding the context of our problem, a variation in the initial position of the obstacle $x_{0}$ or in its speed *v* might have an impact on some or all the defined measures. Also, the presence of abnormal observations will undoubtedly have an important effect on the performance of estimation, as we discuss later on. For these reasons, we will consider that the factors that are likely to have some kind of impact on the three measures of performance are:

- Initial position $x_{0}$ of the obstacle in the tracking problem (Figure 1).
- Speed *v* of the obstacle.
- Amount of biased observations (represented as a percentage of the total number of observations).
- Amount of saturated or missing observations (idem).

Determining to which extent these factors or combinations of them change the performance of Bayesian filters is precisely the core of our study. We will address its concrete implementation in Section 3.5 and Section 3.6, and its results, in Section 4.

### 3.4. Statistical Tools

In this work we employ statistical tools to analyse the performance of Bayesian filters after carrying out exhaustive simulations. These methodologies are useful to derive conclusions about the different aspects of a certain population, seen as different collections of data obtained under particular conditions. We gather these data by simulating sequences of observations, that is, readings from the range sensor, and calculating the corresponding measurements of performance when the filter works on them to estimate the true distance to the obstacle. This is done for as many different conditions as possible combinations of values of the factors mentioned in Section 3.3 exist. Once gathered, the different groups of data are ordered according to such conditions and then analysed from a statistical perspective (please refer to Section 4 for further details). Here, we describe the most suitable tools for our study, as well as how they are implemented.

One of the best-known descriptive and inferential statistical tools is linear regression [50], which, in our case, serves to model the value of a measurement of performance as a function of the concrete conditions of the simulation, given by specific values of the considered factors. The mentioned model would express the performance as a linear combination of the factors plus an error. Since we consider more than one factor in this work, the concrete methodology would be multiple linear regression. Estimating the parameters of the linear combination is usually solved by applying Least Squares Estimation (LSE) [3], which also provides some measurements of the quality of such estimation. Once these parameters are obtained, we can interpret them as the relative influence that each factor has on the performance—the higher the absolute value of the parameter, the greater the influence. However, this is not very reliable since the LSE provides no guarantees on any desirable property of estimators in the general case [3]; thus we will only use this result as a first approximation and perform a more in-depth, rigorous *analysis of variance*.

Analysis of variance (ANOVA) [51] is a statistical methodology that serves to study the differences existing among several groups in a population, where each group corresponds to a subset of a sample that is obtained under given conditions. The variables that explain a specific condition are called factors, which in our case correspond to the ones previously defined in Section 3.3. Also, the aspect of the population under study is referred to as the dependent variable; in our context, it will be one of the measures of performance of the filter. The differences among groups are always studied in terms of their means, thus, ANOVA enables to derive conclusions about the effects that the considered factors are expected to have on the population, that is, on the corresponding measurement of performance.

ANOVA is a statistical method for hypothesis testing. Depending on the number of factors we want to consider, the number and form of the null hypothesis may vary. There exist different kinds of statistical tests that can be embedded in ANOVA, however, the most traditional and the one we will use in this work is the F-test, which relies on the Fisher-Snedecor distribution. Regardless of the number of factors, there are some assumptions that must be met for the validity of the conclusions derived from an F-test [51], namely, the normality of the population data, the *homoscedasticity* of population variances and the independence of observed values. It is easy that the previous conditions are not fully satisfied in a real-world situation. Despite that, ANOVA is relatively robust to violations of these assumptions (please refer to Reference [51] for more details on this issue).

In the context of this work, we perform different ANOVA analyses, as we discuss later on. For this, we consider multiple factors (the ones defined in Section 3.3) that is, we do *n-way* ANOVA. A factor is, in the end, a variable that might produce some behaviour on the population of performance values of the filter, called *main effect*, and it is normally studied for a very reduced set of possible values, for the sake of simplicity. In this work, we will be using two extreme values per factor to cover a wide range of situations. We also take into account the effect produced by the combination of different factors, called *interaction*.

The null hypothesis tested in a one-way ANOVA assumes that the factor under study has no effect in the data. In an *n*-way ANOVA, a null hypothesis per possible group of factors is to be tested, one of each assuming the absence of interaction. This implies that the presence of effect or interaction will hold as long as the null hypothesis is rejected. In order to either accept or reject any hypothesis, some statistic must be calculated; in our case, this will be the *F* statistic. Once calculated, we can perform the corresponding F-test to decide whether the null hypothesis should be rejected or accepted. In this work, we will use a significance level of 0.05 for that purpose (see Reference [51]).

At this point, we could have already obtained a conclusion about the effect that a certain factor of the problem has on the population of performance values gathered from the filter operation. However, it is always a good practice to confirm such conclusion, specially in the case that the considered factor has some distinguishable effect by ANOVA (i.e., in case of rejection of the null hypothesis). This is done by applying some *measure of association strength* to our study. These measures usually represent, in a 0-to-1 scale, the amount of variability of the dependent variable explained by the considered factor. In this work we will be using the *omega squared* measure (${\hat{\omega }}^{2}$) [51]. There is no strict rule to interpret the value of this parameter. As recommended in Reference [52], we will consider that the effect is weak or negligible when ${\hat{\omega }}^{2}$ is close to 0.01 or less, medium or relevant enough when ${\hat{\omega }}^{2}\geq 0.10$, and very strong when ${\hat{\omega }}^{2}\geq 0.25$.

### 3.5. Procedure for the Analysis and Deduction of Conclusions

In order to derive meaningful and unambiguous conclusions it is not sufficient to apply only one *n*-way analysis to our data. A more elaborated procedure based on several applications of ANOVA is required. The main reason is the impossibility of studying the effect of a certain factor or combination of factors in the presence of higher-order interactions involving such factors. To seek both meaningful and concise conclusions, we have devised the procedure formalised in Algorithms A1–A3 (see Appendix A), explained in the following.

The results from an *n*-way ANOVA need to be refined in presence of interactions to interpret effects unambiguously (see Algorithm A1). Such refinement can be done by performing different ANOVA analyses, one of each studying a subset of the population restricted to a specific level of some factor (any other but the one we are interested in). Furthermore, in a higher order analysis this issue should be addressed recursively, since it may arise again in some subset of the population. For this reason, we will always analyse our performance data taking into account higher orders of interaction first. Recall that we can rely on the same analysis for lower orders as long as the higher ones are proven to not have any interaction. In general, a test of effect will hold as long as the involved factors are a subset of a valid higher-order test. The greater the number of interactions, the longer the procedure.

Once the full analysis is complete, we will get conclusions about all the existing factors. Conclusions will always refer to exactly one factor along with a set of restrictions on the others, which would be empty in the case that the conclusion holds for all groups. The union of all conclusions for a factor must cover the entire sample. For instance, in a four-way analysis of a population of values of a performance measure gathered for our problem, using factors *A* (initial position of the obstacle), *B* (amount of missing sensory data), *C* (amount of biased sensory data) and *D* (speed of the obstacle), each one with two possible levels (low and high), complete sets of conclusions for factors could be like the following ones:

- Factor *B* (missing data) has effect on the expected uncertainty of the filter.
- Factor *C* (biased data) has effect on the convergence of the filter given that *B* takes its low value; factor *C* (obstacle speed) has no effect given that *B* takes its high value.
- Factor *D* has no effect on the expected accuracy performance of the filter given that *C* takes its low value; factor *D* has no effect given that *B* takes its low value and *C* its high value; factor *D* has no effect given that both *B* and *C* take their high values.

In the first item, only one conclusion suffices, to explain the effect for any group in the population. Each conclusion in the second item holds for any combination of levels of factors *A* and *D*. The union of the conclusions in the third item also covers all the population groups.

It is always a good practice to check the form of the resulting subset of the population expressed by a conclusion. In this work, we will accept it only if all of the population subsets are normally distributed (or approximately normal) and we will discard the conclusion otherwise (e.g., in case of multimodality). In the latter, we revisit all the necessary analyses, from the lower levels, and *force* some non-existent interactions so that the partition of the population gets more specific and, hopefully, more normal. We also take into account that conclusions should be as concise as possible (see Algorithm A2).

Considering all of the above, we can formally establish the procedure we follow in this work as described in Algorithm A3, which is to be run once per each factor. Since this procedure might be cumbersome, we provide a tree graph that encodes the steps followed by the algorithm for the sake of clarity. In that graph, nodes represent groups of *n* factors involved in a potential *n*-way interaction. In case of no interaction, we use arcs annotated with the factor that will not be considered for the lower order interaction analysis. In case of interaction, we use one or more arcs, each annotated with a specific value of the factor that will be fixed to study the lower interaction or main effect, thus specifying an additional restriction on the population groups. Recall that each of these arcs indicate that a new ANOVA table has been obtained for the studied interaction with the specified restrictions. Finally, the nodes with only one factor indicate that we have reached a valid conclusion on the main effect of the corresponding factor. We also represent more complicated cases such as rejected conclusions due to multimodal data and forced interactions, and provide alternative graphs (below the rejected ones) in order to derive the affected conclusions properly.

**Figure 3:**
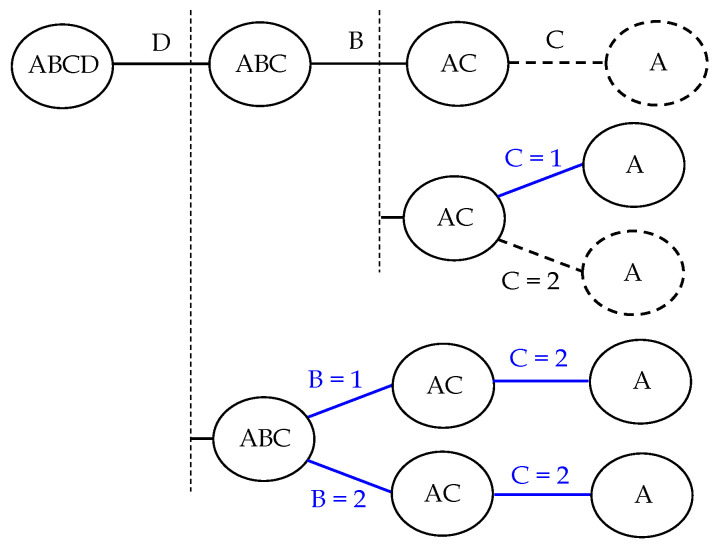
Tree graph for the analysis of the effect of the possible values of factor A (initial position of the obstacle) on the expected accuracy performance of the filter. Dashed nodes and arcs correspond to rejected conclusions due to multimodal populations. Arcs in blue denote decisions on the value of factors based on interactions that are forced by us to get unimodality in the data. Here, “1” and “2” refer to specific levels of the factors.

As an example of this graph, consider the analysis for the population obtained for the expected accuracy performance of the filter, where the four factors mentioned in Section 3.3 have been used, namely, A (initial position of the obstacle), B (amount of missing range data), C (amount of biased range data) and D (speed of the obstacle). The necessary tree graph for the analysis of factor A is shown in Figure 3 (see Section 4.2 for further details on such analysis).

### 3.6. Gathering Data

Our statistical study relies on simulated data in order to reproduce a wide variety of conditions in real environments, to make the number of simulated tests arbitrarily large, and to always have access to the truth state of the system, which in the end is what enables performance measurement and comparison. These simulations are to be performed under the conditions defined by the factors considered in Section 3.3. Thus, there will be one simulation related to each possible combination of their values, and the data for each performance measure will be divided into different groups according to these conditions. For the reasons given in Section 3.4 we are only considering two levels for each factor, which covers their entire range. The concrete values are provided in Table 2.

**Figure 4:**
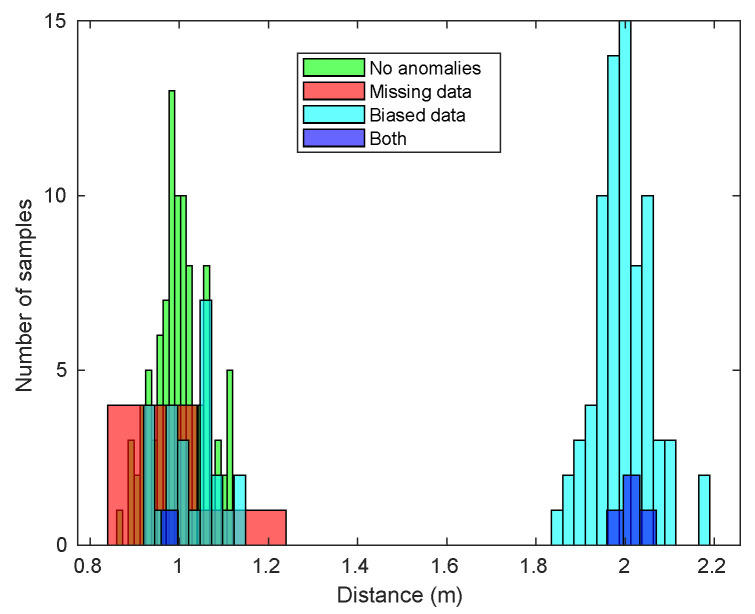
Histograms for sequences of range observation data. These sequences have been obtained from a normal distribution with 1 me of mean; some of them have been corrupted with different combinations of anomalies.

The first step in collecting the performance data from the filter consists in simulating sequences of observations from the range sensor obtained under a particular combination of factor values. In this work we will consider 100 time steps for studying the filter, each of them representing fixed increments of Δt=100 ms (we have chosen that value since it is a suitable sampling time in robotic applications). Each simulated observation is obtained as a random value drawn from a normal distribution with the same mean as the true distance for the corresponding time step and the standard deviation considered for the observation model, that is, σ=0.06 m (see Equation (2)). This vector is then corrupted, if necessary, with biased and/or missing observations placed at random time steps to simulate the anomalies. In these cases, the distribution of observations may differ from a normal one. To illustrate this with an example, we have simulated several sequences of random observations from a normal distribution with 1 meter of mean, and have corrupted some of them with different combinations of anomalies. Figure 4 shows a collection of histograms, each one corresponding to a particular sequence. In these simulations, we assume that the speed of the obstacle is null.

As depicted in Figure 4, when there is an important amount of biased data, the distribution becomes bi-modal, centering in both the original measurement and in the biased one. When there is a high amount of missing data, the lack of observations modifies the shape of the sampled distribution, but there is no reason to affirm that it is not normal. With the combination of the two anomalies, the mentioned effects are also combined: the bias leads to a multi-modal distribution, which is still locally normal despite the lack of data.

Once the necessary observations are simulated, we can infer posterior distributions of the form p$\left(x_{t}|z_{0:t}\right)$, from t=1 to 100, and measure the accuracy and uncertainty of the filter for each *t*. The inference task in the filter is performed by applying the *interface* algorithm [28] (we have used the implementation available in the *Bayes Net Toolbox* (BNT) for Matlab [53]). Since we aim to generate a reasonable amount of data, this simulated experiment is repeated 500 times for each combination of factor values. Algorithm A4 (see Appendix B) details the procedure explained in this paragraph.

Once the necessary performance data have been collected, it is possible to synthesize the three measures of performance we are interested in. Firstly, we have to consider a threshold for the third one, filter convergence (see Section 3.3). If such value is too high, nearly all tests in the data will converge, and, if it is too low, virtually no tests will converge. Under none of these circumstances will we be able to study the filter convergence adequately, thus the proper value must be a tradeoff between the number of tests that we want to converge and the usefulness of the resulting data for our study. After some trials, we have opted for a threshold that allows for at least 45% of converging tests out of a total of 500 for each combination of values of factors. Such a threshold corresponds to a maximum difference of 0.0038 m (3.8 mm) between adjacent accuracies.

In Algorithm A5 (see Appendix B) we illustrate the procedure to synthesize all the measures of performance according to their definition in Section 3.3 from the data collected by Algorithm A4. All the resulting population groups have to contain the same number of elements, that is, be balanced (we will refer to this later on). After discarding those tests that are not converging, the 16 groups for each performance (one for each possible combination of factors) finally have 304 elements each.

**Figure 5:**
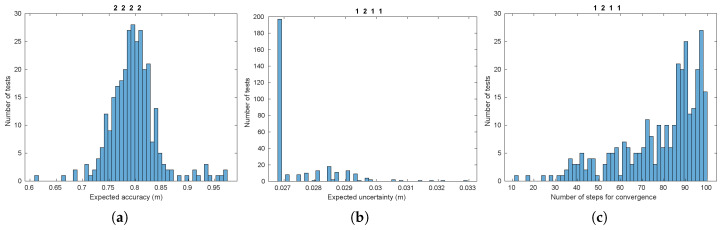
Histograms for some population groups corresponding to the three performances of the filter (refer to Appendix C for the rest). (**a**) Expected accuracy. (**b**) Expected uncertainty. (**c**) Convergence. Each sequence of four numbers in the figure titles represent the concrete combination of values for factors ABCD (see Table 2).

Once the performance measures are obtained, they have to be validated in order to determine whether the necessary requirements to apply our statistical methodologies are fulfilled. Recall that for ANOVA it is necessary that the obtained data for each population group is normally distributed, which is also a desirable condition for the Least Squares Estimator [3]. In Figure 5, histograms for the results of expected accuracy, expected uncertainty and convergence are shown for some representative population groups. A complete account can be found in the figures of Appendix C.

The results in Figure 5a are to some extent similar to the shape of a normal distribution, but the data in Figure 5b have very little variation, that is, they are more similar to a Delta function. This is due to the fact that the estimated uncertainty in a filtering process does not depend on the concrete values of the observations, but on the number of them, among other properties [3]. As a consequence, we will only perceive changes when the number of observations vary or, at least, when the time step they are acquired is different, from test to test. This issue does not prevent us from applying the mentioned statistical methods to these data, as we discuss later on. From the results in Figure 5c, it can be noted that groups in this performance measure present a skewed shape. We have applied some de-skewing processes, but the resulting shapes gets not much better. Fortunately, ANOVA is generally robust to these kind of non-normalities [51].

**Figure 6:**
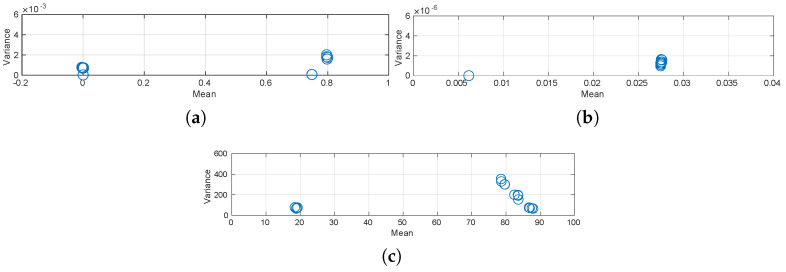
Homoscedasticity of population variances for the performance measures of the range filter. (**a**) Expected accuracy. (**b**) Expected uncertainty. (**c**) Convergence.

Another requirement that must be satisfied is the *homoscedasticity* of variances, that is, the variances of all population groups cannot vary across the means of such groups. We have calculated the mean and variance for each group for all the measurements of performance. The results are depicted in Figure 6. Note that there are 16 points to each graph, one per group. These results show that the required condition is not strongly violated. ANOVA is also relatively robust to mild violations of this criterion [51].

## 4. Results of the Study and Discussion

In this section we state the main hypotheses that we aim to test with our study, discuss the obtained results from the application of rigorous statistical methods, and provide experimental validation of such results in a real environment with a mobile robot.

### 4.1. Statement of Hypotheses

In Section 3.3 we defined the factors that are likely to have some kind of impact on the measures of performance of the filter. These definitions are translated into the hypotheses that we discuss next and, as intuitive affirmations of the behaviour of Bayesian filters, will be confirmed or rejected by the study.

Firstly, it is reasonable to think that, in the context of distance estimation, the conditions of the general tracking problem (i.e., the initial position of the obstacle and its speed) might have some kind of impact on any of the measures of performance. They have a clear influence on the way that the distances under study evolve over time, and therefore could modify the estimation error or even affect the convergence rate.

Observation anomalies must certainly affect the observation model of the filter, since they are not expected nor contemplated in the models of reality it implements. For instance, missing observations produce a lack of data for calculating the filtering estimation given by the posterior distribution $p(x_{t}|z_{0:t})$, and force the filter to work only with prior predictions. In this situation, the estimations could diverge in the case that the transition model was not close enough to reality, increasing without limit the estimation error. If the filter does not diverge, these anomalies would increase progressively the uncertainty of the estimate by the injection of the system motion uncertainty at each step. Following the analytical formulation for convergence reported in Reference [7], in that case the Lyapunov function $V_{k}$, used for defining the closeness of the filter to convergence, becomes larger, therefore making convergence slower.

In the case of bias anomalies, observations still arrive, but the filter is—unknowingly—using a model that is biased w.r.t reality. That perturbation makes the filter to predict observations farther away from actual ones at each affected step, which has consequences on the error in the estimate. Function $V_{k}$ is affected by that increased error, getting larger values which, again, would make convergence slower.

### 4.2. Statistical Analysis

First, we proceed with multiple linear regression. The observed values of each measure of performance *Y* are estimated in this case as a linear combination of the values of the factors *A*, *B*, *C* and *D* (recall Table 2). This method aims to minimize the error between the observed values and the predicted ones $\hat{Y}$, which is expressed as follows:

(7) $$\hat{Y}=\beta_{0}+\beta_{1}A+\beta_{2}B+\beta_{3}C+\beta_{4}D$$

where $\beta_{0},\ldots ,\beta_{4}\in R$.

The results for the three measures of performance of the filter are detailed in Table 3, along with the quality of their estimation, given by the $R^{2}$ statistic [50].

From these results, some interesting conclusions can already be derived by focusing on those parameters with the highest value for each performance. We could state, for instance, that factor C (amount of biased range data) is the most relevant for the filter expected accuracy, implying that the greater the amount of these observations, the worse that accuracy, which is pretty intuitive. Factor B (amount of missing range data) seems to have a clear impact on the uncertainty, that is, a greater number of missing observations hinders the reduction of uncertainty in the filtering process. Lastly, factors B and C are estimated to be relevant for the convergence performance, which is also plausible, since a lower amount of available data usually leads to slower convergence rates (and the same holds for an increase in the amount of biased observations).

Although these conclusions are reasonable and expected, the magnitudes of the coefficients also provide information that is not that obvious. For instance, factors B and C are near 2 orders of magnitude more important than the rest in expected accuracy, and the same holds for factor B in uncertainty. In convergence, these factors share their relevance with the influence of the $\beta_{0}$ parameter. This parameter is not related to any factor, but accounts for the importance of those effects that are not explicitly treated in the analysis, that is, it represents the portion of the performance value that is not explained by the considered factors. This parameter has not a relevant influence on the expected accuracy nor on the uncertainty; however, it is important for convergence, which indicates that there are a number of influences on convergence that are beyond our study of abnormal sensor observations. In this case, the value of $\beta_{0}$ says that, in absence of abnormal observations (represented by factors B and C), the average convergence rate is around approximately 35 steps (see the population groups for convergence in Figure A3).

**Figure A2:**
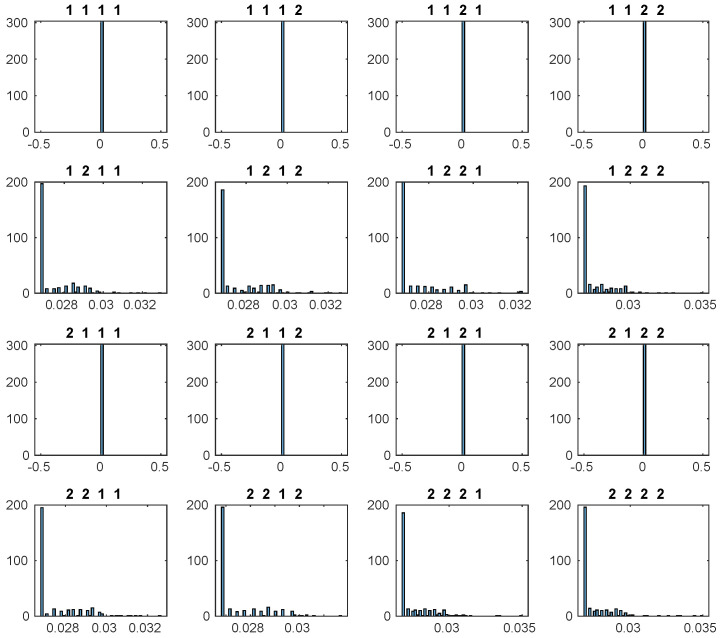
Histograms for all the population groups corresponding to the performance measure of the expected uncertainty of the filter. The horizontal axes represent the expected uncertainty in meters, and the vertical ones, the number of tests.

**Figure A3:**
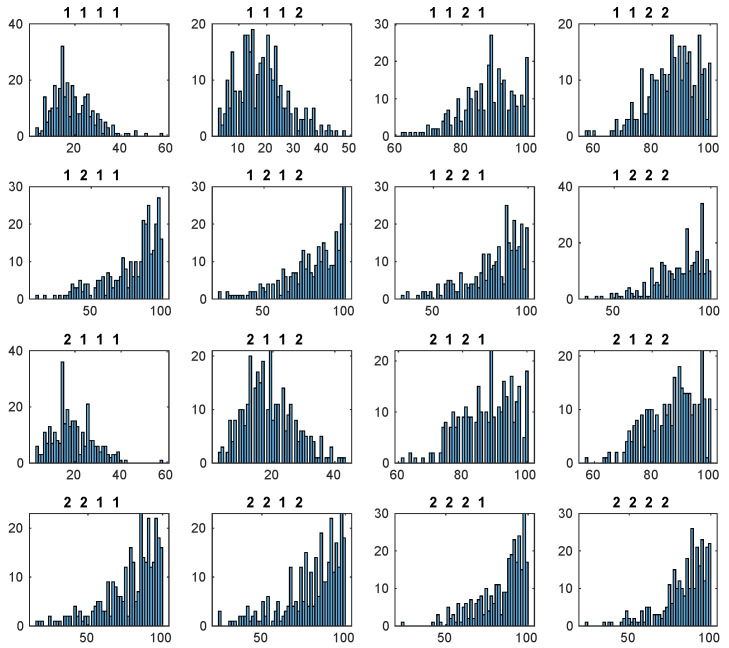
Histograms for all the population groups corresponding to the performance measure of the convergence of the filter. The horizontal axes represent the minimum number of steps $t^{*}$ that lead to convergence, and the vertical ones, the number of tests.

Notice that in this regression analysis there are still information that is not elucidated, like the interaction effects among factors. For a more detailed study we have applied the hypothesis testing procedure explained in Section 3.5. Notice, however, that as shown in Figure A2, all the obtained data for the expected uncertainty of the filter are identical when there are no missing observations (i.e., when factor B takes its low value) for the reasons explained in Section 3.6; thus, it does not make sense to perform ANOVA in that case, but just conclude that none of the factors have any effect in the expected uncertainty of the filter when B = 1.

For the sake of brevity in the following, we focus on the explanation of the final results; the necessary tree graphs for the analyses carried out along with the corresponding ANOVA tables and histograms for obtaining these conclusions are fully reported in Appendix D. In short, we have carried out a total of 12 analyses, 4 per each measure of performance of the filter.

Table 4 provides a complete summary of the conclusions obtained for each factor. Firstly, factors A and D, which define the parameters of the tracking scenario, that is, the initial position of the obstacle and its speed, are statistically assessed not to affect any measure of performance, regardless of their values. This is compatible with the results obtained by the multiple linear regression method, and it is plausible, since there is no reason to consider that the concrete values of the gathered distances (or the rate at which they vary) have any undesirable effect on the steady-state performances, providing that they reproduce reality adequately (i.e., they are not obtained under anomalous conditions).

Regarding abnormal observations, missing sensory readings (factor B), usually provoked by the presence of obstacles with transparent or absorbent surfaces or by conditions of extreme lighting, have a negative impact on all the performances in most cases; more concretely, as the occurrences of this anomaly increase (B = 2), the performances get worse, but a relevant and not obvious conclusion of this study is that accuracy is affected by the presence of missing readings only if these kind of data occurs along with biased data, although the impact is not very strong.

In the case of the expected uncertainty of the filter, an increase in the percentage of missing readings always leads to a higher uncertainty. As predicted by the linear regression method, only this factor has relation with the uncertainty; the main reason is the fact that the filter uncertainty can be reduced as more observations are available at the time of inference, under certain conditions.

Another non-obvious conclusion on the influence of missing data is that the convergence of the filter is only affected by an increase of missing observations in the case that these readings are *not* combined with any biased sensor readings, otherwise the effect of missing data being negligible. In other words, biased observations produce an influence that “hides” the one of missing data in the convergence of the filter; the very effect of biased readings is sufficient to seriously deteriorate the convergence (see Table 4).

Biased observations (factor C), which are often provoked by excessive reflections of the waves emitted by sensors on the scene, also have an important and negative effect on the performances with the exception of the filtering uncertainty, which does not depend on the concrete values of the readings but on the number of them, as discussed before. For the case of the expected accuracy of the filter, an increase in the percentage of biased readings always leads to a much worse accuracy, regardless of the remaining conditions. A result that is not so straightforward is that filter convergence is only affected by biased data when these are not combined with missing ones: the very effect of missing observations is strong enough to noticeably worsen the convergence rate, again “hiding” the effects of biased data. In conclusion, once that one of these kinds of anomalous sensory data are present, the effect of the other is negligible in convergence, although biased data has worse effect in the magnitude of convergence.

ANOVA does not provide conclusions about the effects on the standard deviation; in the end, they are considered less relevant than the ones produced on the means of the factors; however, we have analysed them as well. In this case, variations in the value of factor B (amount of missing readings) always lead to relevant changes on the standard deviation, even when it is proved that there is no effect on the mean. Regarding factor C (amount of biased observations), the differences are not that important in most cases, with the exception of the expected accuracy performance of the filter. Lastly, the remaining factors do not have any noticeable impact on the standard deviation in any case.

As we have proved, only factors B and C, which correspond to the amount of abnormal sensory data, have some kind of effect on the steady-state performances of the filter. Such anomalous sensory readings are not infrequent in real scenarios where mobile robots typically operate as discussed in Section 3.2. For instance, navigation in large corridors may well lead to a high amount of missing sensory data, due to the fact that the maximum detection range of the on-board sensors is systematically exceeded in the longest direction. Unfortunately, this is not the only situation that could lead to the same issue, and there are, in fact, many of them (e.g., navigation under conditions of extreme infrared radiation, navigation nearby highly reflective surfaces, etc.). Biased readings are also common in these kinds of sensors, and are usually due to particular features of the scene (e.g., presence of geometrically challenging surfaces, such as corners, or highly reflective ones as well, etc.). There are also some situations where both kinds of abnormal sensory data can be combined (although not simultaneously). For example, in a scene with a high presence of thin obstacles, such as chair legs, range sensors may produce both biased and missing readings alternately, sometimes due to a high number of reflections and other times due to sudden detections of free space, respectively.

An inadequate value in any of the measures of performance has a negative impact on the operation of a mobile robot that we have assessed statistically in the previous analysis and also accompanied by general magnitudes to be expected. More concretely, essential tasks such as navigation, localization and mapping may result seriously compromised. For instance, an increase in the amount of biased sensory data worsens the expected accuracy of the filter, and, in this situation, the pose of the operating mobile robot could not be estimated properly, biasing it as well. Similarly, a less accurate perception of the scene may affect the mapping of such environment, and this affects, in turn, subsequent navigation, compromising the robotic operation. Abnormal observations such as missing readings have a negative impact on the expected uncertainty: the higher the number of these observations, the higher the filtering uncertainty. In extreme conditions, this may result in useless distance estimations in the scope of an obstacle tracking scenario, or in localization or mapping problems, since an estimation with high uncertainty cannot be considered to solve any of these problems. Finally, the presence of a high amount of either missing or biased sensory readings negatively affects the convergence of the filter. A slow convergence rate could, for instance, limit the maximum navigation speed, since it would not be safe for a robot to operate within the scene relying on highly uncertain or inaccurate distance estimations. In the case that the speed could not be limited, this issue would lead to a poor localization and mapping, due to the low quality of the estimations.

### 4.3. Real Experiment

**Figure 7:**
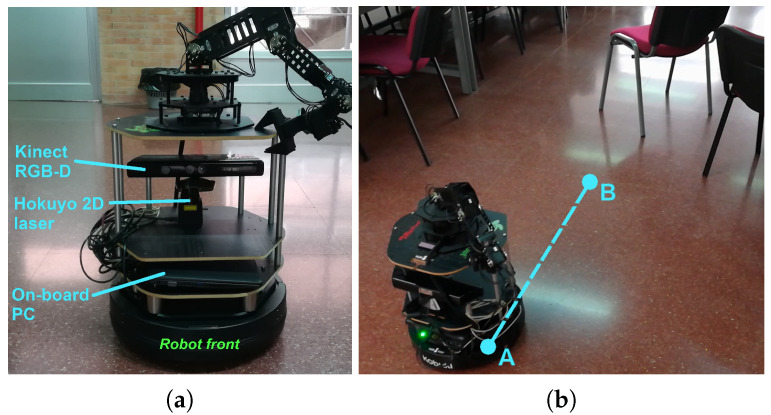
Experimental setup. (**a**) Frontal view of the CRUMB robot with its devices. (**b**) Indoor scenario used for the experiment. Here, the robot moves at a constant speed from point A to B towards a chair with gray legs placed in front of it, which produces a number of anomalies.

In this section we illustrate the conclusions obtained in Section 4.2 with a experiment in a real environment. For that, we have used a mobile platform, the CRUMB robot [54], which is based on a version of the *Turtlebot-2* that uses a two-wheeled *Kobuki* platform [55]. This mobile robot is endowed, among others, with two range sensors relying on infrared radiation, namely, a *Hokuyo URG-04-LX* 2-D laser [45] and a *Kinect V1* RGB-D camera [46,47], whose main features were already included in Table 1. The CRUMB robot is also equipped with an on-board netbook PC with an Intel Celeron N2840 at 2.16 GHz and 2 GB DDR3 that runs Ubuntu 14.04 with ROS [56]. A picture of this robot can be seen in Figure 7a.

The experiment takes place in the indoor scenario shown in Figure 7b. This setup aims to reproduce the conditions of the general obstacle tracking problem studied in this work (recall Figure 1). In this case, the robot moves at a constant speed from point A to B, while facing a static obstacle that is to be detected by the range sensors on board. We will only deal with those measurements gathered in the very direction of movement, corresponding to the gray chair leg that is closest to the robot.

**Figure 8:**
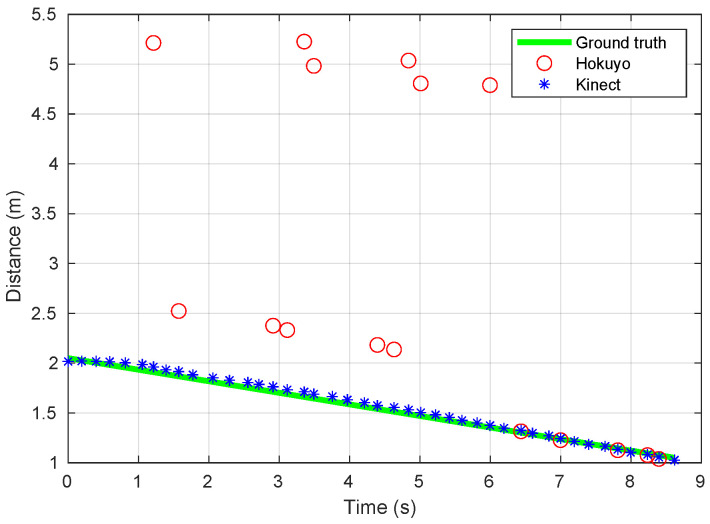
Range measurements obtained by the *Hokuyo* and the *Kinect* sensors during the experiment in Figure 7, along with the ground-truth distances.

The CRUMB robot covers in this experiment a distance of 1 m. This has been measured manually in the real scene, as well as the ground-truth distance to the obstacle, which is 2.05 m when the robot is placed at point A and 1.05 m when it is at point B. Also, the measured speed is 0.116 m/s. The obtained sensory measurements from both sensors along with the ground-truth distances are shown in Figure 8.

The gathered data show that the *Kinect* sensor has worked reasonably well during the experiment and that no anomalies have affected it. In contrast, the *Hokuyo* laser rangefinder has suffered from abnormal conditions up to a point that its observations are rarely correct: the obtained measurements are mostly biased and or missing, corresponding to the extreme position of these factors in the statistical study of Section 4.2. The obstacle is, in the end, a reflective surface that may have provoked the reflection of the central laser beam over another nearby chair legs (see Figure 7b) leading to a larger distance than the actual one. Also, this beam may have been reflected to an empty area, leading, as a result, to a missing observation. The reason why the *Kinect* sensor is not affected by the same situation is probably due to the fact that its mesurement principle, although based on infrared radiation, is different.

The three sources of data present in Figure 8 are needed for comparing this experiment to the conclusions of the statistical study—the measures of the filtering performance could not be obtained without knowing the ground-truth distances; also, we would not be able to extract any conclusion on the effects of abnormal conditions on such performance without a fault-free situation.

We have used the Bayesian filter in the form of a DBN, as explained in Section 3.1, and have calculated the performance measures as detailed in Section 3.3. We have modified the parameters of both the observation and transition models of the filter (Equations (2) and (4), respectively) so that they adapt to the concrete conditions of the real experiment. In particular, the standard deviation of sensory measurements has been set to σ=0.08 m, since it represents the average accuracy of both the *Hokuyo* and *Kinect* sensors (see Table 1). Also, the speed in the transition model has been set to v=−0.116 m/s, where the negative sense is due to the fact that it is the robot which moves in this case, and not the obstacle. Also, the value of Δt is not constant and has to be modified in each iteration of the filter. In our case, this value has a mean of 0.21 s and a standard deviation of 0.04 s.

**Figure 9:**
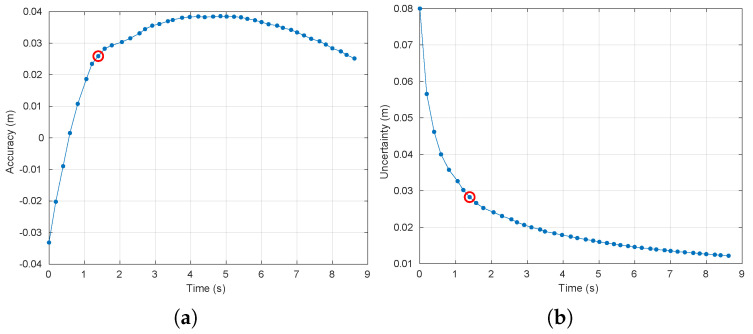
Evolution over time of the measures of filtering performance for the case of use of the *Kinect* sensor. The red circle indicates the instant of convergence (after 8 filtering steps). (**a**) Accuracy. (**b**) Uncertainty.

**Figure 10:**
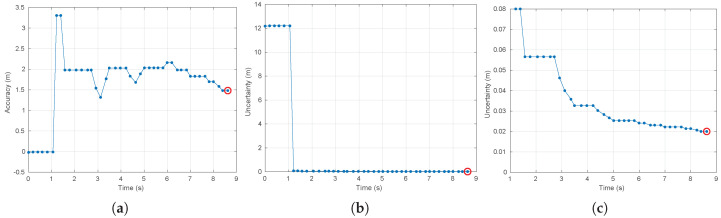
Evolution over time of the measures of filtering performance for the *Hokuyo* sensor. The red circle indicates the instant of convergence (43 filtering steps). (**a**) Accuracy. (**b**) Uncertainty. (**c**) Zoomed view (vertically) of the uncertainty.

Figure 9 and Figure 10 show the accuracy and uncertainty performance measures over time as well as the convergence achieved in the case of use of the Kinect and Hokuyo sensors, respectively. Furthermore, the steady-state measures of performance are collected in Table 5.

With these results, we can see that all the measures of performance are worse for the case of the *Hokuyo* sensor, which was affected by both biased and missing data anomalies, even if we consider their evolution over time. These results also allow us to validate some of the most important conclusions of our study, which were reported in Table 4. Firstly, the combined presence of anomalies in the experiment with the *Hokuyo* rangefinder leads to a much worse expected accuracy compared to the fault-free situation of the *Kinect* sensor: biased readings are sufficient to deteriorate this performance, regardless of the remaining conditions. Second, the combination of anomalies in the real experiment provokes an increase on the expected uncertainty, which is also compatible with the obtained conclusions, since the sole presence of missing readings is expected to worsen this performance. Finally, the abnormal conditions in the real setup also lead to a much slower convergence, which is again compatible with the statements of the study, since only the presence of one of the anomalies is enough to produce this effect.

## 5. Conclusions

In this work, we have studied the impact of abnormal observations on the performance of generic Bayesian filters. For that, we have addressed Bayesian filtering inference from a generalist perspective, by using the paradigm of Dynamic Bayesian Networks. We have modelled this generic Bayesian filter taking into account the features of the most common robotics sensors, have analysed their main limitations and have studied those factors that are likely to affect the filter performance. Different simulated experiments with diverse conditions have been designed, and novel and relevant conclusions have been obtained from their use with rigorous statistical methods. Also, these conclusions have been validated in a real situation.

Our results show that the parameters of the tracking problem considered for our study do not have any relation with the performance of Bayesian filters. In contrast, the increase of the amount of abnormal sensory data, that is, missing and biased observations, generally affects all the performances negatively. The combination of both kinds of anomalous data worsens the expected accuracy of the filter, while only missing observations are capable of increasing the filtering uncertainty. Lastly, one of the conclusions that was not expected before conducting the statistical analyses is that the convergence performance is seriously affected by both kinds of anomalous observations separately, and that their combination does not lead to a worse convergence rate in case of an already deteriorated situation.

There are some tasks that we plan to address in future works. The conclusions derived from our study currently rely on a set of factors that can be expanded to include a wider variety of sensors, filtering parameters and modes of robotic operation. The impact of variations on all of these aspects would be studied regarding filtering performance. Also, we plan to study such performance in the scope of more general models of Bayesian estimation, such as hybrid models like the Switching Kalman Filter [28], which can also be implemented within the framework provided by Dynamic Bayesian Networks.

## Figures and Tables

**Table 1:** Main features of common range sensors in mobile robotics. Here, the accuracy reported is the worst-case error w.r.t the true distance, and the type of sensor includes the number of dimensions and the measurement principle.

Model Name	Type	Detectable Range	Accuracy
Devantech SRF05 [43]	1D ultrasonic	0.01 to 4 m	4 cm
Sharp GP2Y0A02YK [44]	1D triangulation-based IR	0.2 to 1.5 m	10 cm
Hokuyo URG-04LX-UG01 [45]	2D laser-based	0.06 to 4 m	12 cm
Microsoft Kinect V1 [46,47]	3D structured-light	0.5 to 4 m	4 cm
SwissRanger SR4000 [48]	3D ToF camera	0.1 to 5 m	10 mm

**Table 2:** Factors influencing the performance of Bayesian range filters along with the concrete values that they can take, including both scenario parameters and sensor anomalies. In this work, we will be using “1” and “2” to refer to the low and high values of the factors respectively.

Factor	Meaning	Low Value “1”	High Value “2”
A	Initial distance to obstacle ($x_{0}$)	1 m	2 m
B	Amount of missing range data	0%	95%
C	Amount of biased range data	0%	75%
D	Obstacle speed (*v*)	0 m/s	0.2 m/s

**Table 3:** Multiple linear regression coefficients obtained for the three measures of performance of the range filter and quality of their estimations ($R^{2}$). Maximum values are highlighted in bold.

Factor	Parameter	Expected Accuracy	Expected Uncertainty	Convergence
-	$\beta_{0}$	−0.0119	0.0061	34.6577
A	$\beta_{1}$	−0.0001	0.0000	0.1509
B	$\beta_{2}$	0.0253	**0.0225**	**29.6187**
C	$\beta_{3}$	**1.0316**	0.0000	**48.3876**
D	$\beta_{4}$	−0.0064	0.0000	0.3762
-	$R^{2}$	0.9945	0.9943	0.5563

**Table 4:** Summary of the conclusions obtained for the effect that each factor has on the performances of the filter. Again, “1” and “2” stand for the low and high levels of the factors, respectively. See Table 2 for the numerical values of the factors. Here, μ and σ represent the mean and standard deviation of the factor. The symbol “—” denotes no effect on the mean, which is indicated along with its value, and “↑” represents an increase in that value, which is accompanied in this case by the difference between means at each extreme (the high one minus the low one). In each cell, the extreme values of the standard deviation are also reported.

Factor	Expected Accuracy			Expected Uncertainty		Convergence		
A (obstacle position)	C = 1	μ(—): $-6.82\times 10^{-4}$ m σ: 0.02, 0.02				B = 1	C = 1	μ(—): 19 steps σ: 8, 8
	C = 2	B = 1	μ(—): 0.75 m σ: 0.009, 0.009	B = 1	μ(—): 0.006 m σ: 0, 0		C = 2	μ(—): 87 steps σ: 8, 8
		B = 2	μ(—): 0.80 m σ: 0.041, 0.044	B = 2	μ(—): 0.028 m σ: 0.001, 0.001	B = 2	μ(—): 81 steps σ: 16, 16	
B (% of missing data)	C = 1	μ(—): 0.003 m σ: 0.006, 0.027		μ(↑): 0.02 m σ: 0, 0.001		C = 1	μ(↑): 60 steps σ: 8, 18	
	C = 2	μ(↑): 0.05 m σ: 0.009, 0.040				C = 2	μ(—): 85 steps σ: 8, 14	
C (% of biased data)	B = 1	μ(↑): 0.75 m σ: 0.006, 0.009		B = 1	μ(—): 0.006 m σ: 0, 0	B = 1	μ(↑): 68 steps σ: 8, 8	
	B = 2	μ(↑): 0.80 m σ: 0.027, 0.042		B = 2	μ(—): 0.028 m σ: 0.0011, 0.0012	B = 2	μ(—): 81 steps σ: 18, 14	
D (obstacle speed)	C = 1	μ(—): $-6.82\times 10^{-4}$ m σ: 0.02, 0.02				B = 1	C = 1	μ(—): 19 steps σ: 8, 8
	C = 2	B = 1	μ(—): 0.75 m σ: 0.009, 0.009	B = 1	μ(—): 0.006 m σ: 0, 0		C = 2	μ(—): 87 steps σ: 8, 8
		B = 2	μ(—): 0.80 m σ: 0.042, 0.042	B = 2	μ(—): 0.028 m σ: 0.001, 0.001	B = 2	μ(—): 81 steps σ: 15, 16	

**Table 5:** Steady-state measures of filtering performance for the sensors used in the real experiment. The expected accuracy and uncertainty were calculated taking into account the last 4 steps of the filter.

Measure	Kinect	Hokuyo
Expected accuracy	0.0268 m	1.5599 m
Expected uncertainty	0.0124 m	0.0205 m
Steps for convergence	8	43

**Figure A1:**
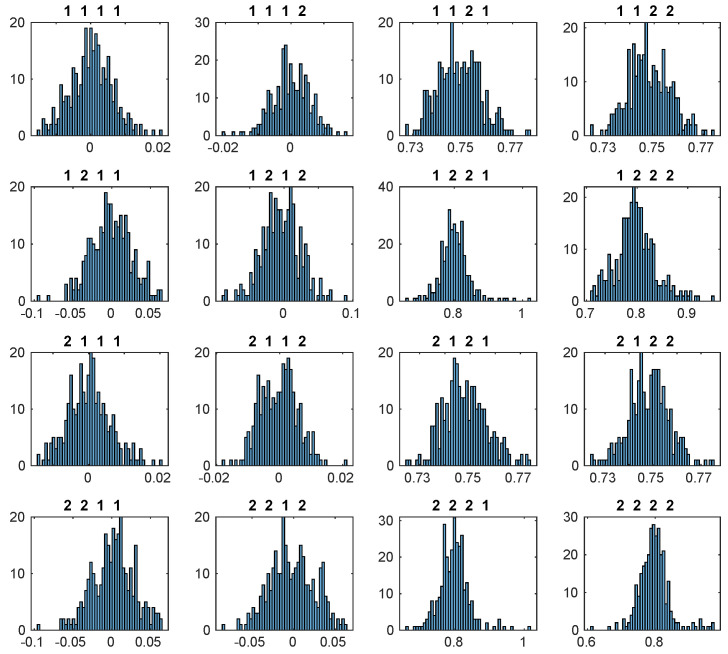
Histograms for all the population groups corresponding to the performance measure of the expected accuracy of the filter. Each sequence of four numbers in the figure titles represent the concrete combination of values for factors ABCD (see Table 2). The horizontal axes represent the expected accuracy in meters, and the vertical ones, the number of tests. There are 16 different groups with 304 tests each.

**Figure A4:**
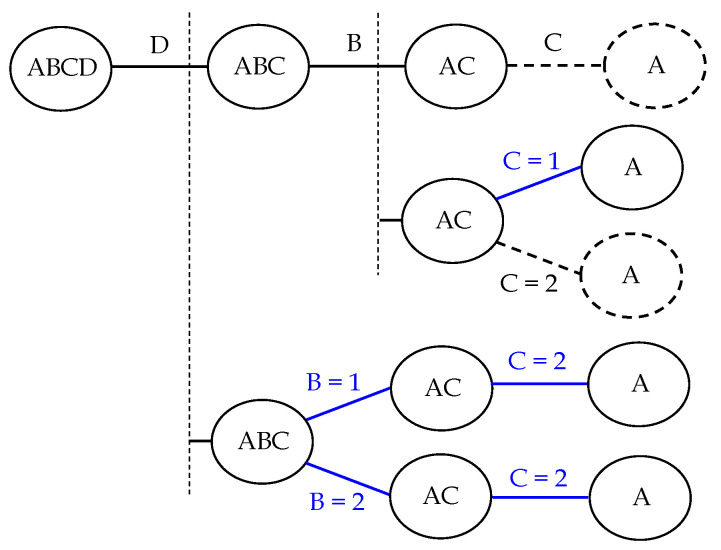
Tree graph for the analysis of the effect of factor A on the expected accuracy performance of the filter. Dashed nodes and arcs correspond to rejected conclusions due to multimodal populations. Arcs in blue denote decisions on the value of factors based on interactions that are forced by us to get unimodality in the data.

**Figure A5:**
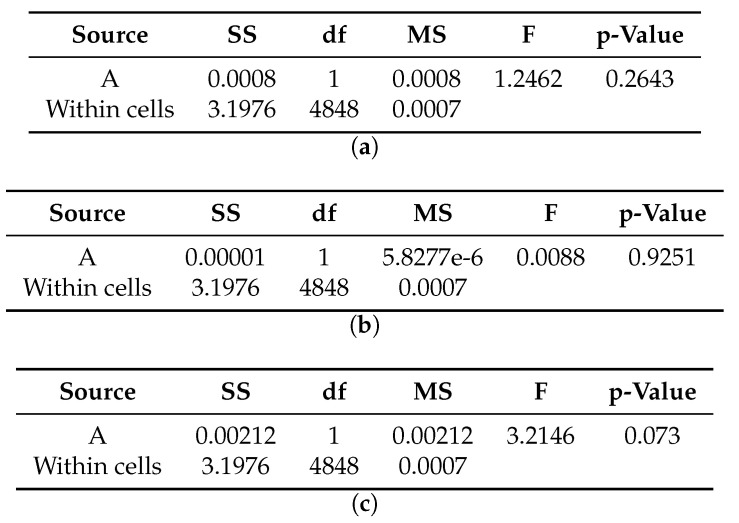
ANOVA tables for the analysis of the effect of factor A (initial distance of the object to the sensor) on the expected accuracy of the filter. (**a**) One-way ANOVA for factor A given C = 1. (**b**) One-way ANOVA for factor A given B = 1 and C = 2. (**c**) One-way ANOVA for factor A given B = 2 and C = 2.

**Figure A6:**
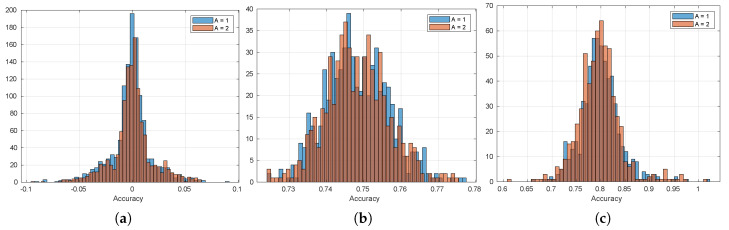
Histograms for the conclusions about the effect of factor A on the expected accuracy performance, represented for the two levels of such factor with additional restrictions on the population. (**a**) Factor C = 1. (**b**) Factor B = 1 and factor C = 2. (**c**) Factor B = 2 and factor C = 2.

**Figure A7:**
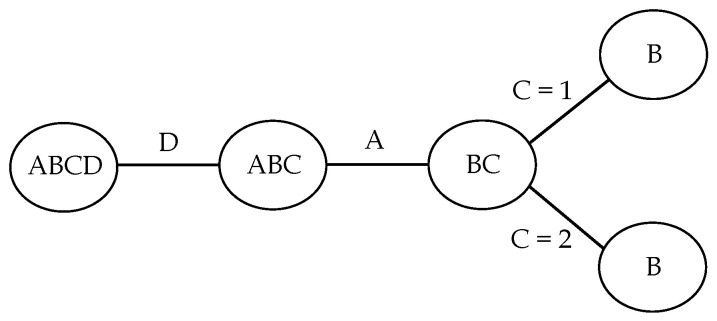
Tree graph for the analysis of the effect of factor B on the expected accuracy performance.

**Figure A8:**
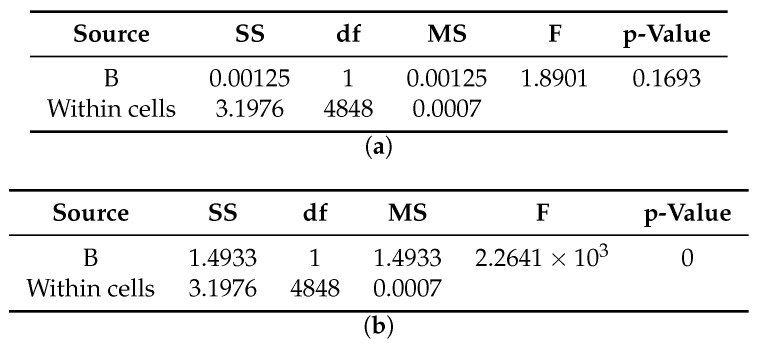
ANOVA tables for the analysis of the effect of factor B (amount of missing observations) on the expected accuracy. (**a**) One-way ANOVA for factor B given C = 1. (**b**) One-way ANOVA for factor B given C = 2.

**Figure A9:**
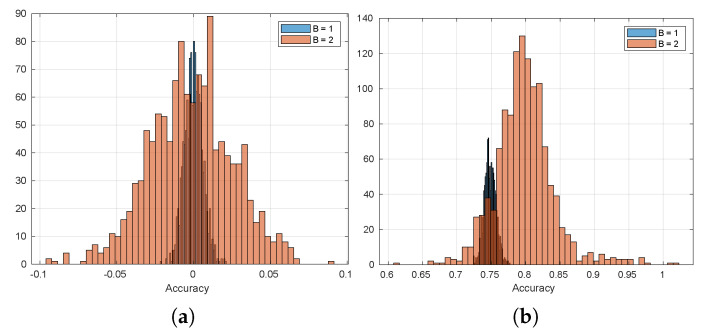
Histograms for the conclusions about the effect of factor B on the expected accuracy performance, represented for the two levels of such factor with additional restrictions on the population. (**a**) Factor C = 1. (**b**) Factor C = 2.

**Figure A10:**
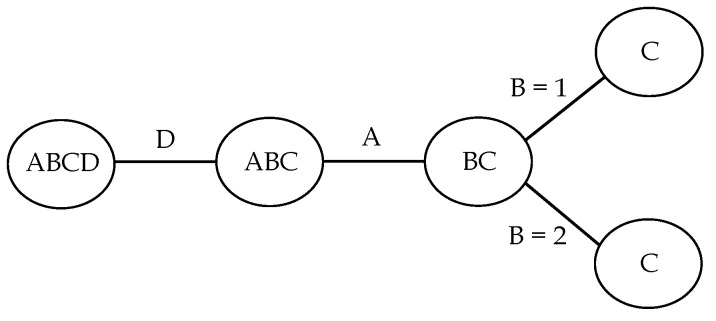
Tree graph for the analysis of the effect of factor C on the expected accuracy performance.

**Figure A11:**
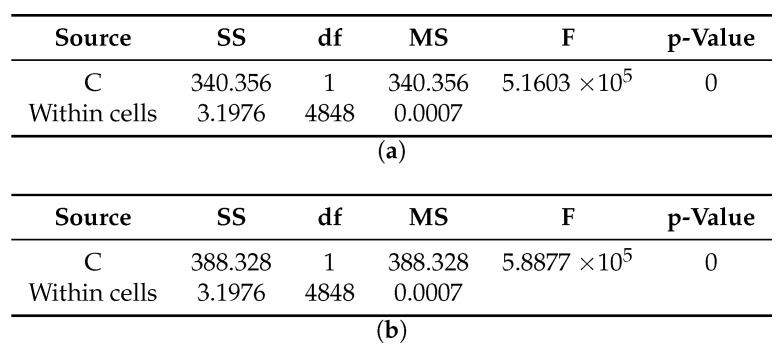
ANOVA tables for the analysis of the effect of factor C (amount of biased observations) on the expected accuracy. (**a**) One-way ANOVA for factor C given B = 1. (**b**) One-way ANOVA for factor C given B = 2.

**Figure A12:**
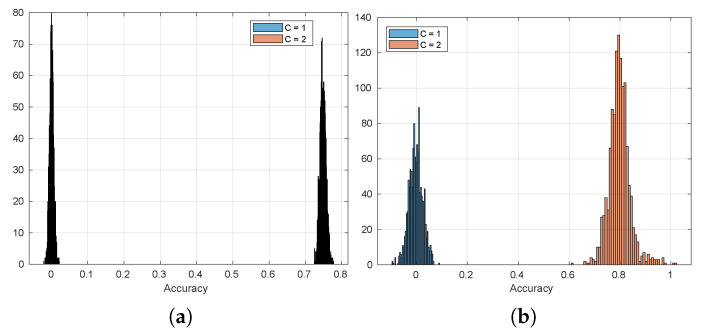
Histograms for the conclusions about the effect of factor C on the expected accuracy performance, represented for the two levels of such factor with additional restrictions on the population. (**a**) Factor B = 1. (**b**) Factor B = 2.

**Figure A13:**
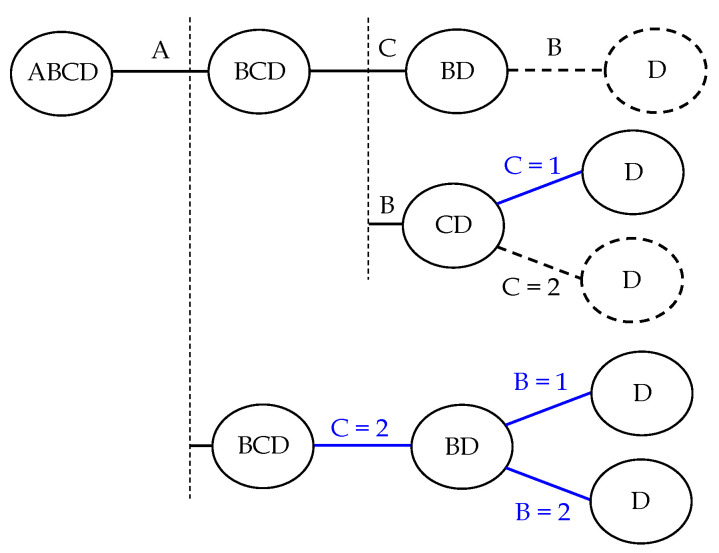
Tree graph for the analysis of the effect of factor D on the expected accuracy performance. Dashed nodes and arcs correspond to rejected conclusions due to multimodal population. Arcs in blue denote decisions on the value of factors based on forced interactions.

**Figure A14:**
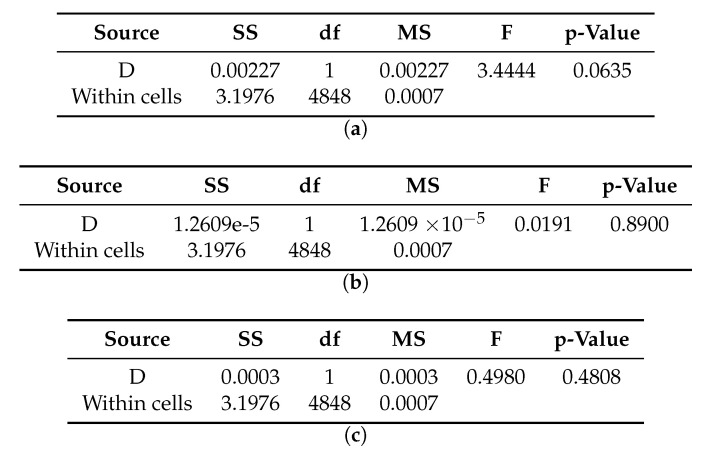
ANOVA tables for the analysis of the effect of factor D (speed of the obstacle) on the expected accuracy. (**a**) One-way ANOVA for factor D given C = 1. (**b**) One-way ANOVA for factor D given B = 1 and C = 2. (**c**) One-way ANOVA for factor D given B = 2 and C = 2.

**Figure A15:**
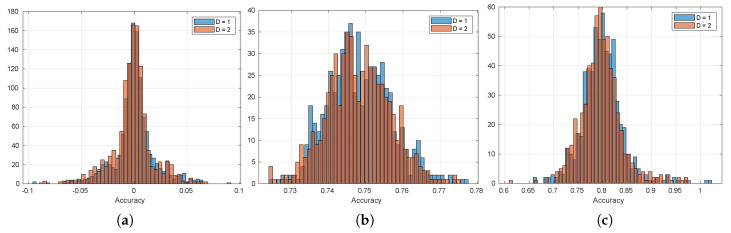
Histograms for the conclusions about the effect of factor D on the expected accuracy performance, represented for the two levels of such factor with additional restrictions on the population. (**a**) Factor C = 1. (**b**) Factor B = 1 and factor C = 2. (**c**) Factor B = 2 and factor C = 2.

**Figure A16:**
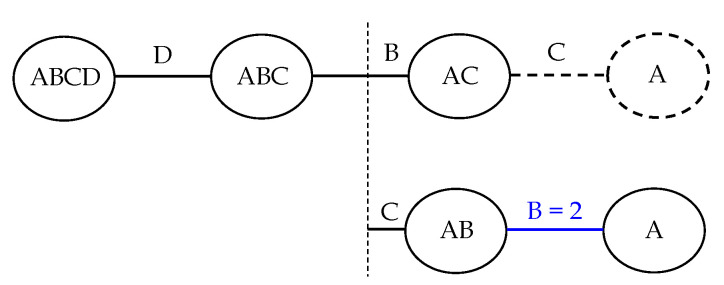
Tree graph for the analysis of the effect of factor A on the expected uncertainty performance. The dashed node and arc correspond to a rejected conclusion due to multimodal population. The arc in blue represents a decision on the value of factor B based on a forced interaction.

**Figure A17:**
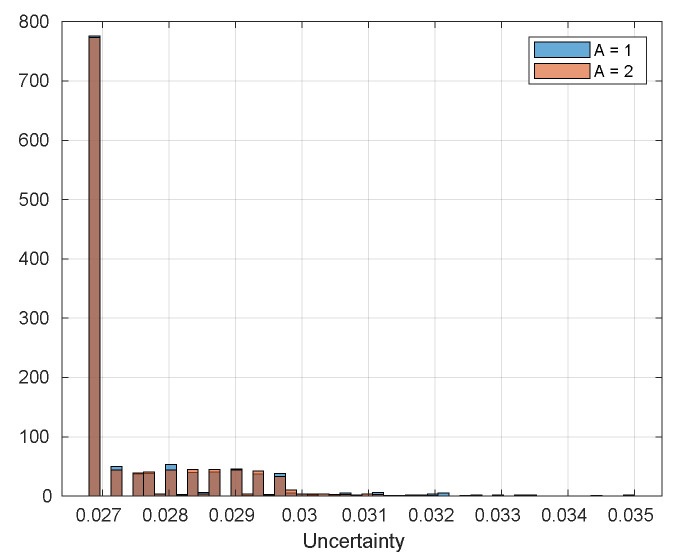
Histograms for the conclusion of the effect of factor A on the expected uncertainty performance when B = 2, represented for the two levels of factor A.

**Figure A18:**
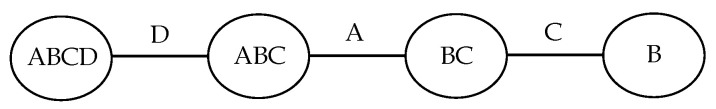
Tree graph for the analysis of the effect of factor B on the expected uncertainty performance.

**Figure A19:**
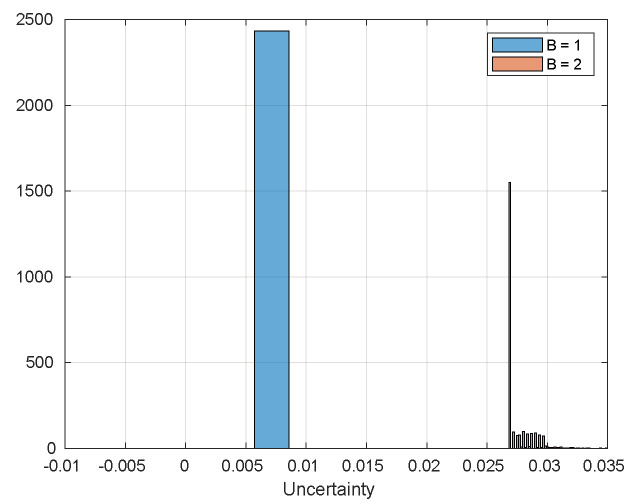
Histograms for the conclusion of the effect of factor B on the expected uncertainty performance, represented for the two levels of the factor.

**Figure A20:**
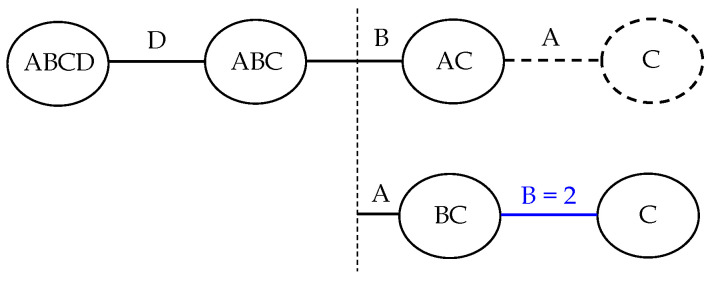
Tree graph for the analysis of the effect of factor C on the expected uncertainty performance. The dashed node and arc correspond to a rejected conclusion due to multimodal population. The arc in blue represents a decision on the value of factor B based on a forced interaction.

**Figure A21:**
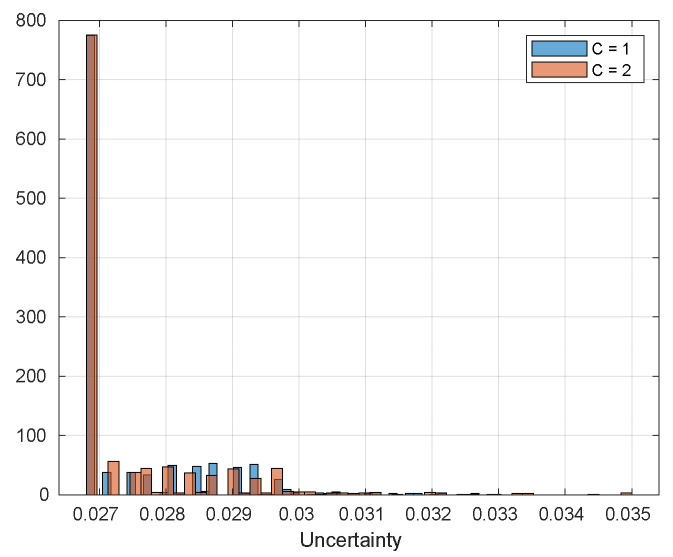
Histograms for the conclusion of the effect of factor C on the expected uncertainty performance when B = 2, represented for the two levels of factor C.

**Figure A22:**
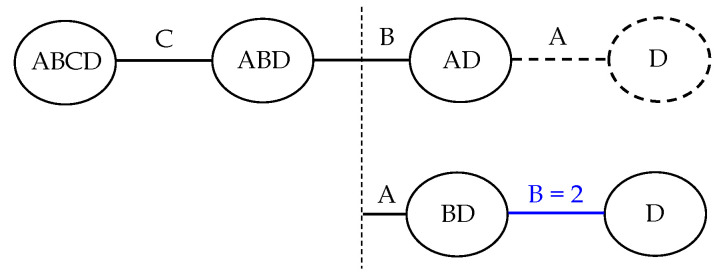
Tree graph for the analysis of the effect of factor D on the expected uncertainty performance. The dashed node and arc correspond to a rejected conclusion due to multimodal population. The arc in blue represents a decision on the value of factor B based on a forced interaction.

**Figure A23:**
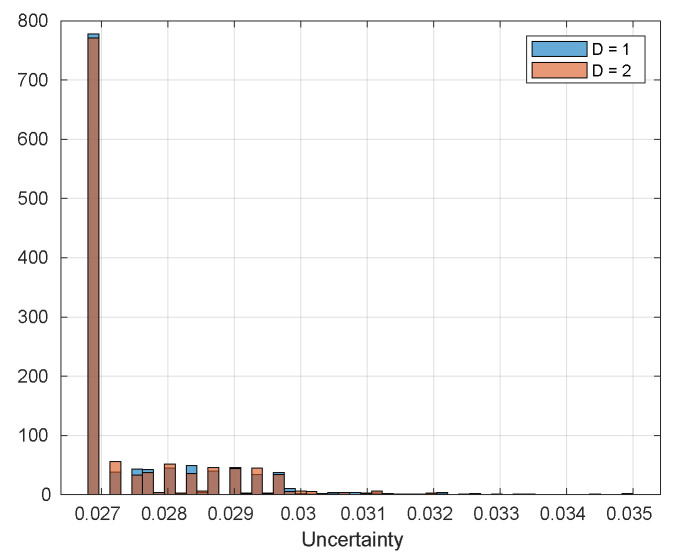
Histograms for the conclusion of the effect of factor D on the expected uncertainty performance when B = 2, represented for the two levels of factor D.

**Figure A24:**
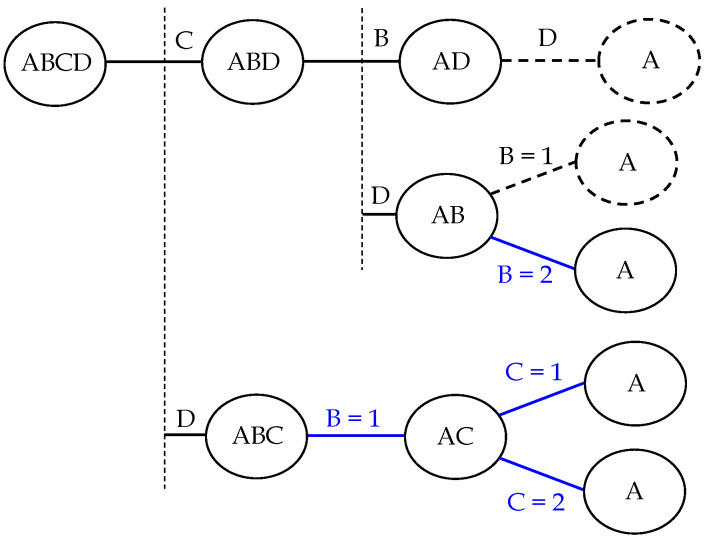
Tree graph for the analysis of the effect of factor A on the convergence performance. Dashed nodes and arcs correspond to rejected conclusions due to multimodal population. Arcs in blue denote decisions on the value of factors based on forced interactions.

**Figure A25:**
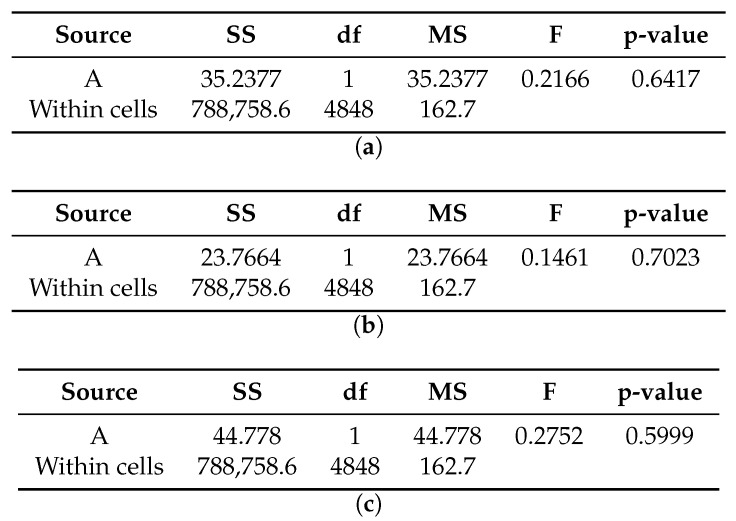
ANOVA tables for the analysis of the effect of factor A (initial position of the obstacle) on the convergence. (**a**) One-way ANOVA for factor A given B = 1 and C = 1. (**b**) One-way ANOVA for factor A given B = 1 and C = 2. (**c**) One-way ANOVA for factor A given B = 2.

**Figure A26:**
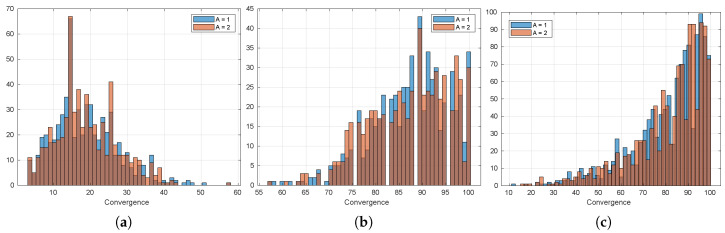
Histograms for the conclusions about the effect of factor A on the convergence performance, represented for the two levels of such factor with additional restrictions on the population. (**a**) Factor B = 1 and factor C = 1. (**b**) Factor B = 1 and factor C = 2. (**c**) Factor B = 2.

**Figure A27:**
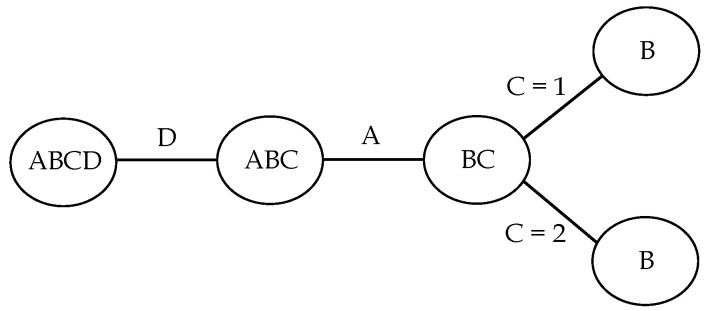
Tree graph for the analysis of the effect of factor B on the convergence performance.

**Figure A28:**
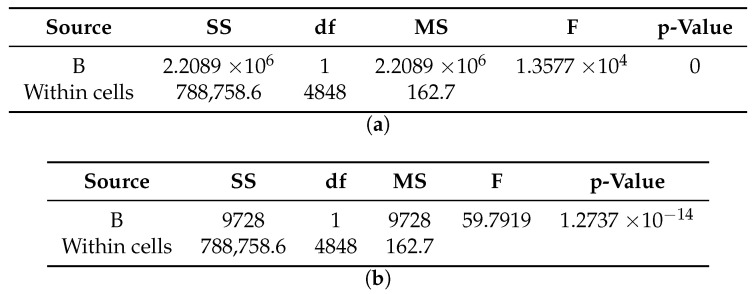
ANOVA tables for the analysis of the effect of factor B (amount of missing observations) on the convergence. (**a**) One-way ANOVA for factor B given C = 1. (**b**) One-way ANOVA for factor B given C = 2.

**Figure A29:**
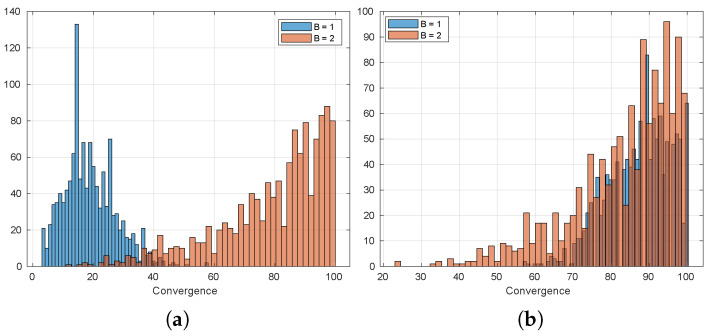
Histograms for the conclusions about the effect of factor B on the convergence performance, represented for the two levels of such factor with additional restrictions on the population. (**a**) Factor C = 1. (**b**) Factor C = 2.

**Figure A30:**
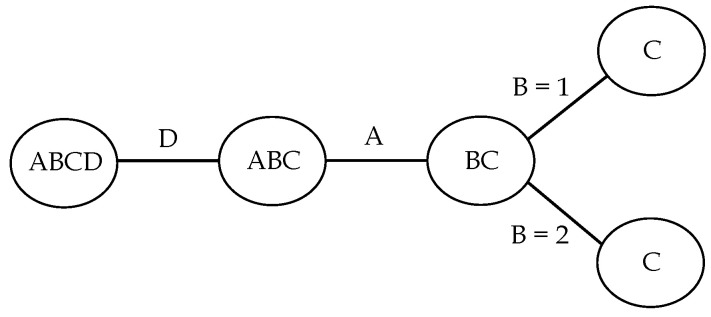
Tree graph for the analysis of the effect of factor C on the convergence performance.

**Figure A31:**
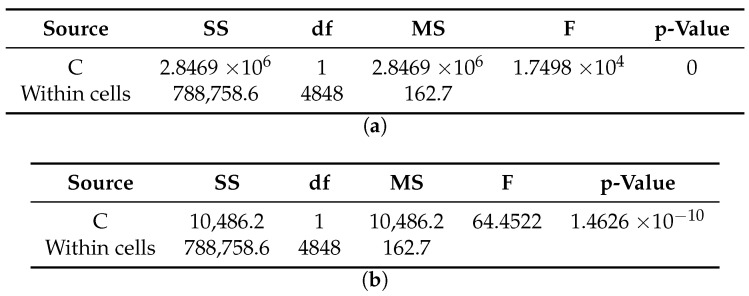
ANOVA tables for the analysis of the effect of factor C (amount of biased observations) on the convergence. (**a**) One-way ANOVA for factor C given B = 1. (**b**) One-way ANOVA for factor C given B = 2.

**Figure A32:**
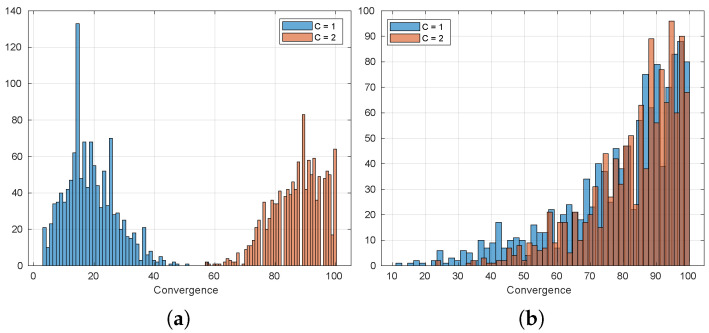
Histograms for the conclusions about the effect of factor C on the convergence performance, represented for the two levels of such factor with additional restrictions on the population. (**a**) Factor B = 1. (**b**) Factor B = 2.

**Figure A33:**
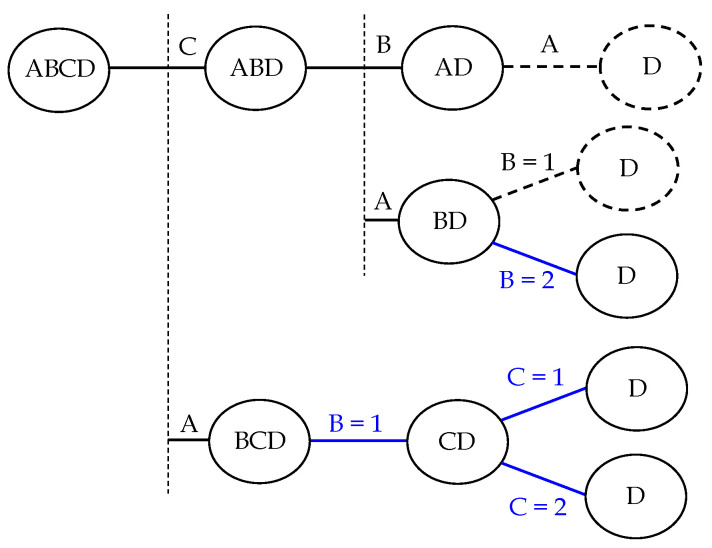
Tree graph for the analysis of the effect of factor D on the convergence performance. Dashed nodes and arcs correspond to rejected conclusions due to multimodal population. Arcs in blue denote decisions on the value of factors based on forced interactions.

**Figure A34:**
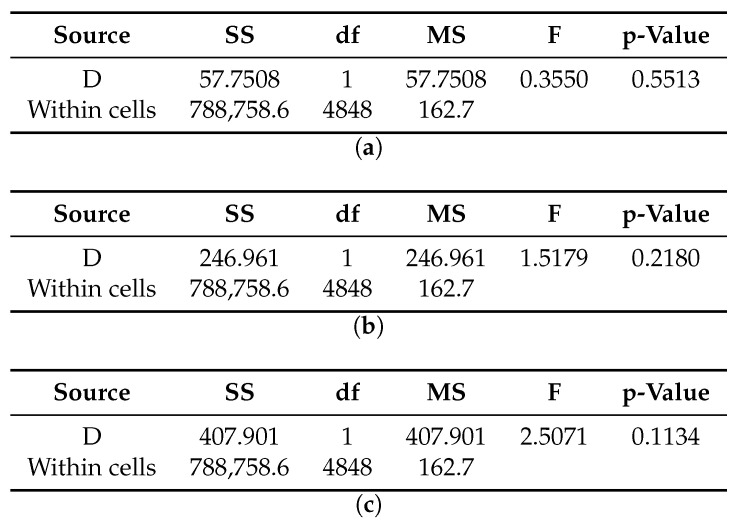
ANOVA tables for the analysis of the effect of factor D (speed of the obstacle) on the convergence. (**a**) One-way ANOVA for factor D given B = 1 and C = 1. (**a**) One-way ANOVA for factor D given B = 1 and C = 2. (**a**) One-way ANOVA for factor D given B = 2.

**Figure A35:**
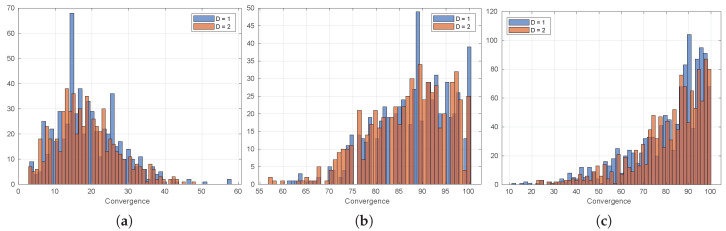
Histograms for the conclusions about the effect of factor D on the convergence performance, represented for the two levels of such factor with additional restrictions on the population. (**a**) Factor B = 1 and factor C = 1. (**b**) Factor B = 1 and factor C = 2. (**c**) Factor B = 2.

## Appendix A. Algorithms for the Deduction of Conclusions

**Algorithms A1.***testInteractions*(*n*, *s*, *X*, F, *T*)
**input:** *n*: level of interaction *s*: significance level *X*: factor of interest F: set of all considered factors *T*: ANOVA table **output:** S: set of factors involved in an *n*-way interaction **main:** 1: L ← set of possible combinations of *n* factors from F containing *X* 2: S ←*∅* 3: **for**i=1 to |L| **do** 4: $p_{i}$ ← *p*-value from interaction test involving factors in $L_{i}\subset L$ using *T* 5: **if** $p_{i}<s$ **then** 6: ${\hat{\omega }}^{2}$ ← omega squared value for interaction involving factors in $L_{i}$ 7: **if** ${\hat{\omega }}^{2}\geq 0.10$ **then** 8: S ← $S\cup (L_{i},p_{i})$ 9: **end if** 10: **end if** 11: **end for** 12: S ← search for the $L_{i}$ in S with the lowest $p_{i}$ 13: **return** S

**Algorithm 2.***forceInteraction*(*n*, *a*, *f*, *X*, **F**, *error*, S)
**input:** *n*: level of interaction *a*: number of possible interactions *f*: number of attempts *X*: factor of interest F: set of all considered factors error: boolean value indicating the impossibility of obtaining valid conclusions S: set of pairs of factors (auxiliary variable) **output:** S: set of factors involved in an *n*-way interaction *Z*: factor for the study of interaction involving factors in S Updates of variables *n*, *a*, *f*, error and S **subroutines:** generateInteractions(n,X,F): $\;\{\left(S_{i},Z_{i}\right)\}\leftarrow$ set of all possible pairs of *n*-way interactions $S_{i}\subset F$ and factors $Z_{i}\in S_{i}\backslash \left\{X\right\}$ $\;S\leftarrow \{\left(S_{i},Z_{i}\right)\}$ **return** S getInteraction(f,S): (S,Z)← search for *f*-th pair in S **return** (S,Z) **main:** 1: f←f+1 2: **if** (n=1 **or** f=a+2) **and** n≤|F| **then** 3: n←n+1 4: **else if** n>|F| **then** 5: error←true 6: **end if** 7: **if** (f=1 **or** f=a+2) **and** error=false **then** 8: S←generateInteractions(n,X,F) 9: a←|S| 10: f←2 11: **if** a=0 **then** 12: error←true 13: **end if** 14: **end if** 15: **if** error=false **then** 16: (S,Z)←getInteraction(f,S) 17: **else** 18: S←∅ 19: Z←∅ 20: **end if** 21: **return** (S,Z,n,a,f,error,S)

**Algorithm 3.***ANOVA*(*s*, *X*, **F**, **Y**, **R**)
**input:** *s*: significance level *X*: factor of interest F: set of all considered factors in the population Y: population data indexed by levels of factors in F (i.e., $Y=\{Y_{11\ldots 1},Y_{11\ldots 2},\ldots \}$) R: set of restrictions on the entire population **output:** C: set of conclusions for factor *X* **main:** 1: C←∅, p←∅, S←∅, f←0, a←(−2), n←|F|, error←false (initialization) 2: T← perform an *n*-way ANOVA over population Y and store its corresponding table 3: **while**error=false**and** conclusions in C do not cover the entire population **do** 4: **if** n>1 **then** 5: **if** f=0 **then** 6: S←testInteractions(n,s,X,F,T) 7: **end if** 8: **if** S=∅ **then** 9: n←n−1 10: **else** 11: **if** f=0 **then** 12: Z← choose one factor in S\{X} 13: **end if** 14: l← number of levels of factor *Z* 15: **for** i=1 to *l* **do** 16: D← subset of the population Y verifying Z=i 17: R←R∪{Z=i} 18: C←C∪ANOVA(s,X,S\{Z},D,R) 19: R←R\{Z=i} 20: **end for** 21: **if** conclusions in C do not cover the entire population **then** 22: (S,Z,n,a,f,error,S)← *forceInteraction(n, a, f, X, F, error, S)* 23: **end if** 24: **end if** 25: **else** 26: **if** population is not normal for all levels of factor *X* given R **then** 27: (S,Z,n,a,f,error,S)← *forceInteraction(n, a, f, X, F, error, S)* 28: **else** 29: p← *p*-value from main effect test for factor *X* using *T* 30: ${\hat{\omega }}^{2}\leftarrow$ omega squared value for main effect *X* 31: **if** p<s **and** ${\hat{\omega }}^{2}\geq 0.10$ **then** 32: C← C ∪ { Factor *X* has effect given R} 33: **else** 34: C← C ∪ { Factor *X* has no effect given R} 35: **end if** 36: **end if** 37: **end if** 38: **end while** 39: **return** C

## Appendix B. Algorithms for Gathering Simulated Data

**Algorithm 4.** *dataCollection*
**output:** $E_{ABCD}$: data concerning filter accuracy, indexed by combinations of values of factors $U_{ABCD}$: data concerning filter uncertainty, indexed by combinations of values of factors **main:** 1: n←500 (number of experiments for each combination of factors) 2: T←100 (number of total time steps considered) 3: Δt←0.1 (sampling time in seconds) 4: σ←0.06 (standard deviation in meters considered for the observation model) 5: $E_{ABCD}\leftarrow \emptyset$ 6: $U_{ABCD}\leftarrow \emptyset$ 7: **for each** possible combination ABCD of values of factors **do** 8: **for** *i*=1 to *n* **do** 9: g←∅ (vector of ground-truth distances) 10: z←∅ (vector of observations) 11: **for** *t*=0 to *T* **do** 12: $x_{0}\leftarrow$ initial distance to obstacle (from value of factor A) 13: v← obstacle speed (from value of factor D) 14: $g\left(t\right)\leftarrow x_{0}+t\cdot v\Delta t$ 15: z(t)← random value drawn from distribution $N(g\left(t\right),\sigma^{2})$ 16: **end for** 17: **if** B=2 **then** // *absorption anomaly* 18: z← corrupt current vector z with 95% of empty observations at random positions 19: **end if** 20: **if** C=2 **then** // *bias anomaly* 21: z← corrupt current vector z by adding 1 m to 75% of non-empty positions randomly 22: **end if** 23: $\hat{\mu }\leftarrow \emptyset$ (vector of estimated distances) 24: $\hat{\sigma }\leftarrow \emptyset$ (vector of standard deviations for estimated distances) 25: **for** t=1 to *T* **do** 26: p$(x_{t}|z_{0:t})\leftarrow$ calculate filter posterior by using the interface algorithm (see Section 3.1) 27: $\hat{\mu }\left(t\right)\leftarrow$ mean of the normal distribution represented by p$\left(x_{t}|z_{0:t}\right)$ 28: $\hat{\sigma }\left(t\right)\leftarrow$ standard deviation of the normal distribution represented by p$\left(x_{t}|z_{0:t}\right)$ 29: $E_{ABCD}\left(i,t\right)\leftarrow \hat{\mu }\left(t\right)-g\left(t\right)$ 30: $U_{ABCD}\left(i,t\right)\leftarrow \hat{\sigma }\left(t\right)$ 31: **end for** 32: **end for** 33: **end for** 34: **return** ($E_{ABCD}$, $U_{ABCD}$)

**Algorithm 5.***performanceData*($E_{ABCD}$), $U_{ABCD}$
**input:** $E_{ABCD}$: population data for filter accuracy, indexed by combinations of values of factors $U_{ABCD}$: population data for filter uncertainty (indexed as above) **output:** ${\bar{E}}_{ABCD}$: population data for the expected accuracy performance (indexed as above) ${\bar{U}}_{ABCD}$: population data for the expected uncertainty performance (indexed as above) $C_{ABCD}$: population data for the convergence performance (indexed as above) **main:** 1: n←500 (number of tests in each population group) 2: C←∅ (set of ordered indices of converging tests) 3: f←∅ (vector of filtered accuracy values) 4: ${\bar{e}}_{ABCD}\leftarrow \emptyset$ (temporary population data for expected accuracy) 5: ${\bar{u}}_{ABCD}\leftarrow \emptyset$ (temporary population data for expected uncertainty) 6: $c_{ABCD}\leftarrow \emptyset$ (temporary population data for convergence) 7: m←0 8: $m_{s}\leftarrow 100$ 9: **for each** possible combination ABCD of values of factors **do** 10: **for** *i*=1 to *n* **do** 11: ${\bar{e}}_{ABCD}\left(i\right)\leftarrow$ average of accuracy values in vector $E_{ABCD}\left(i\right)$ for the last 10 time steps 12: ${\bar{u}}_{ABCD}\left(i\right)\leftarrow$ average of uncertainty values in vector $U_{ABCD}\left(i\right)$ for the last 10 time steps 13: f← apply a 5-th order median filter to vector $E_{ABCD}\left(i\right)$ 14: **if** $\exists t^{*}:\left|f\left(t\right)-f\left(t-1\right)\right|\leq 0.0038\;\left(\forall t\geq t^{*}\right)$ **then** 15: C←C∪{i} 16: $c_{ABCD}\left(i\right)\leftarrow t^{*}$ 17: **end if** 18: **end for** 19: **for** j=1 to |C| **do** 20: i← *j*-th element of *C* 21: ${\bar{E}}_{ABCD}\left(j\right)\leftarrow {\bar{e}}_{ABCD}\left(i\right)$ 22: ${\bar{U}}_{ABCD}\left(j\right)\leftarrow {\bar{u}}_{ABCD}\left(i\right)$ 23: $C_{ABCD}\left(j\right)\leftarrow c_{ABCD}\left(i\right)$ 24: **end for** 25: m←|C| 26: **if** $m<m_{s}$ **then** 27: $m_{s}\leftarrow m$ 28: **end if** 29: **end for** 30: Discard tests randomly such that $|{\bar{E}}_{ABCD}\left|=\right|{\bar{U}}_{ABCD}\left|=\right|C_{ABCD}|=m_{s}$ for any ABCD 31: **return** (${\bar{E}}_{ABCD}$, ${\bar{U}}_{ABCD}$, $C_{ABCD}$)

## Appendix C. Population Groups for the Performances of the Filter

In this appendix we show the histograms corresponding to the population groups obtained for the three measures of performance of the filter. Recall that there are 16 groups with 304 tests each for each performance. Results for expected accuracy, uncertainty and convergence are depicted in Figure A1, Figure A2 and Figure A3, respectively.

## Appendix D. Full Report of the Obtained Results

In this appendix we show all the tree graphs, ANOVA tables and population histograms that have been used during the procedure explained in Section 3.5 and that constitute the basis of the derived conclusions, presented in Section 4.2. These results are organised here by each measure of performance and each factor. For the sake of brevity, we omit those ANOVA tables appearing in the process that lead to invalid, multimodal conclusions.

### Appendix D.1. Expected Accuracy Performance

Firstly, we begin with the expected accuracy performance. There is a common first step to all the analyses consisting in the obtention of a four-way ANOVA for the population data. This is shown in Table A1.

**Table A1:** Four-way ANOVA table for the expected accuracy performance.

Source	SS	df	MS	F	p-Value
A	0.0000	1	0.0000	0.0237	0.8775
B	0.7041	1	0.7041	1067.5699	0.0000
C	727.8939	1	727.8939	1,103,603.2161	0.0000
D	0.0020	1	0.0020	3.0079	0.0829
AxB	0.0000	1	0.0000	0.0005	0.9822
AxC	0.0020	1	0.0020	3.0027	0.0832
AxD	0.0001	1	0.0001	0.1927	0.6607
BxC	0.7904	1	0.7904	1198.4044	0.0000
BxD	0.0013	1	0.0013	1.9418	0.1635
CxD	0.0005	1	0.0005	0.7927	0.3733
AxBxC	0.0020	1	0.0020	2.9629	0.0853
AxBxD	0.0000	1	0.0000	0.0264	0.8709
AxCxD	0.0002	1	0.0002	0.3474	0.5556
BxCxD	0.0005	1	0.0005	0.6824	0.4088
AxBxCxD	0.0001	1	0.0001	0.0855	0.7700
Within cells	3.1976	4848	0.0007		

The tree graph for the case of the expected accuracy population with factor A is depicted in Figure A4 and the ANOVA tables used during the process are collected in Figure A5. Taking into account these results, we can state a complete set of conclusions for factor A (again, we use the reduced notation for the values of factors shown in Table 2):

- Factor A has no effect on the expected accuracy of the filter given that C = 1.
- Factor A has no effect given that B = 1 and C = 2.
- Factor A has no effect given that B = 2 and C = 2.

This summarizes into the fact that the initial position of the obstacle has no relevant influence on the accuracy, regardless of the value of the remaining factors. Here we have not used the omega squared measure because none of the effects nor interactions were considered important. Note as well that all the interactions considered in Figure A4 have been forced by us in order to get unimodality. In Figure A6, histograms of the population data are shown for this performance at different levels of factor A, according to the conclusions previously stated. Recall that ANOVA only studies the differences among the groups means; in this case it is shown that such difference is barely noticeable. A secondary effect that can be pointed out here is the fact that an increase in the percentage of missing observations (factor B) leads to a higher variance in the expected accuracy that can be obtained by the filter (comparing Figure A6b,c), which can be of importance in a practical range sensing application.

Now we turn our attention to the case of factor B (the anomaly of missing range measurements due, for instance, to absortions on particular surfaces). The tree graph for this analysis is shown in Figure A7 and the corresponding ANOVA tables appear in Figure A8. This time, only two conclusions are needed to explain the data:

- Factor B has no effect on the expected accuracy of the filter given that C = 1.
- Factor B has effect given that C = 2.

In this case we are dealing with positive effects and interactions; in order to confirm them, we have calculated the necessary omega squared values. For the BxC interaction (see Table A1) we get that ${\hat{\omega }}^{2}=0.1975$, and, for the B main effect with C = 2, ${\hat{\omega }}^{2}=0.3175$, thus, the ANOVA results can be accepted. The above conclusions imply that the amount of missing observations in a range sensor has effect on the filter accuracy only in the case that the amount of biased observations is high. This can be viewed in the histograms shown in Figure A9 for this factor and its restrictions. More specifically, the percentage of missing observations has no effect when there are no biased observations, although the variance in this performance increases noticeably when it is higher (Figure A9a). On the other hand, the presence of missing observations leads to a worse expected accuracy and also increases the variance of the expected accuracy of the filter (Figure A9b).

Regarding factor C (amount of biased observations, produced for instance by reflections), the tree graph is depicted in Figure A10 and the ANOVA tables employed in the process, in Figure A11. The obtained conclusions for this factor are:

- Factor C has effect on the expected accuracy of the filter given that B = 1.
- Factor C has effect given that B = 2.

The interaction in the four-way ANOVA that leads to these conclusions is again BxC (see Table A1), and we provided an omega squared value for it before. In this case, omega squared values are also necessary for assessing both main effects of factor C; these are ${\hat{\omega }}^{2}=0.9907$ for the case B = 1 and ${\hat{\omega }}^{2}=0.9918$ for the case B = 2, thus confirming the strength of the effects. As predicted by the linear regression method, the percentage of biased observations has a high influence on the filter accuracy, regardless of the value of the remaining factors. This can be noticed in the histograms of Figure A12. When the presence of biased observations is high, the accuracy is much worse in general. The only influence of factor B consists in the increase of the variance of this performance measure (this can be noticed comparing Figure A12a,b, although it is only a secondary effect).

Lastly, we address the analysis for factor D (speed of the obstacle). The corresponding tree graph is shown in Figure A13 and the ANOVA tables generated during the process appear in Figure A14. Given these results, the complete set of conclusions for factor D are:

- Factor D has no effect on the expected accuracy of the filter given that C = 1.
- Factor D has no effect given that B = 1 and C = 2.
- Factor D has no effect given that B = 2 and C = 2.

In this case there is no need to check any omega squared value, since there are no interactions nor main effects. Also, note that all the interactions appearing in Figure A13 have been forced by us to get unimodal data. These conclusions allow us to ensure that the speed of the obstacle has no relevant influence on the filter accuracy, regardless of the value of the remaining factors. This can be seen in the histograms of Figure A15, where the only difference relies on the fact that the accuracy mean and variance increase when factors B and C take their highest values; this has nothing to do with the impact of factor D.

### Appendix D.2. Expected Uncertainty Performance

Now we proceed to the analysis of the expected uncertainty performance. Following the same procedure as before, the first step consists in performing a four-way ANOVA for the population data in this case (see Table A2), which will be the basis for the subsequent analyses.

**Table A2:** Four-way ANOVA table for the expected uncertainty performance.

Source	SS	df	MS	F	p-Value
A	1.3281 $\times \;10^{-7}$	1	1.3281 $\times \;10^{-7}$	0.2015	0.6535
B	0.5550	1	0.5550	842,141.6088	0.0000
C	2.5223 $\times \;10^{-8}$	1	2.5223 $\times \;10^{-8}$	0.0383	0.8449
D	7.8373 $\times \;10^{-10}$	1	7.8373 $\times \;10^{-10}$	0.0012	0.9725
AxB	1.3281 $\times \;10^{-7}$	1	1.3281 $\times \;10^{-7}$	0.2015	0.6535
AxC	9.9627 $\times \;10^{-7}$	1	9.9627 $\times \;10^{-7}$	1.5118	0.2189
AxD	1.7472 $\times \;10^{-6}$	1	1.7472 $\times \;10^{-6}$	2.6514	0.1035
BxC	2.5223 $\times \;10^{-8}$	1	2.5223 $\times \;10^{-8}$	0.0383	0.8449
BxD	7.8373 $\times \;10^{-10}$	1	7.8373 $\times \;10^{-10}$	0.0012	0.9725
CxD	6.3747 $\times \;10^{-8}$	1	6.3747 $\times \;10^{-8}$	0.0967	0.7558
AxBxC	9.9627 $\times \;10^{-7}$	1	9.9627 $\times \;10^{-7}$	1.5118	0.2189
AxBxD	1.7472 $\times \;10^{-6}$	1	1.7472 $\times \;10^{-6}$	2.6514	0.1035
AxCxD	1.1712 $\times \;10^{-7}$	1	1.1712 $\times \;10^{-7}$	0.1777	0.6733
BxCxD	6.3747 $\times \;10^{-8}$	1	6.3747 $\times \;10^{-8}$	0.0967	0.7558
AxBxCxD	1.1712 $\times \;10^{-7}$	1	1.1712 $\times \;10^{-7}$	0.1777	0.6733
Within cells	0.00319	4848	6.5899 $\times \;10^{-7}$		

We now begin the analysis by addressing the case of factor A (initial position of the obstacle). The corresponding tree graph is depicted in Figure A16 and the only ANOVA needed for this factor is shown in Table A3. In this case, a special situation arises. As shown in Figure A2, all the obtained data for the performance are identical when there are no missing observations (i.e., when factor B takes its low value) for the reasons explained in Section 3.6. Under these circumstances, it does not make sense to perform any ANOVA; we simply conclude that none of the factors have any effect when B = 1, since no change in the population distribution takes place. Taking this into account and the obtained results for the case B ≠ 1, the complete set of conclusions for factor A is:

- Factor A has no effect on the expected uncertainty of the filter given that B = 1.
- Factor A has no effect given that B = 2.

These results imply that the initial position of the obstacle does not have any influence on the uncertainty of the filter, as shown in the histograms of Figure A17. We will omit, from now on, all the histograms related to the case B = 1, since they are all identical.

**Table A3:** One-way ANOVA table for factor A given B = 2.

Source	SS	df	MS	F	p-Value
A	2.6562 $\times \;10^{-7}$	1	2.6562 $\times \;10^{-7}$	0.4031	0.5255
Within cells	0.00319	4848	6.5899 $\times \;10^{-7}$		

For factor B (amount of missing observations), the resulting tree graph is shown in Figure A18. In this case no extra ANOVA tables are necessary, since factor B has effect with strength ${\hat{\omega }}^{2}=0.9943$, which can be derived directly from Table A2. This result implies that the amount of missing observations from the sensor has an important impact on the expected uncertainty of the filter. As explained before, the number of available observations in a filtering process influence the uncertainty of the posterior distribution. In this case, an increase in the percentage of missing observations lead to a greater uncertainty, as shown in the histograms of Figure A19, regardless of the value of the rest of the factors.

Now we turn our attention to the analysis of factor C (amount of biased observations). The corresponding tree graph is depicted in Figure A20 and the necessary ANOVA is shown in Table A4. With these results, the conclusions derived for this factor are:

- Factor C has no effect on the expected uncertainty of the filter given that B = 1.
- Factor C has no effect given that B = 2.

This means that the abnormality of biased observations in the sensor (e.g., reflections) do not modify the filter uncertainty in any case, which can be seen in the histograms of Figure A21. This is due to the fact that uncertainty does not depend on the concrete values of the observations but on the number of them, as we discussed before.

**Table A4:** One-way ANOVA table for factor C given B = 2.

Source	SS	df	MS	F	p-Value
C	5.0446 $\times \;10^{-8}$	1	5.0446 $\times \;10^{-8}$	0.0766	0.7820
Within cells	0.00319	4848	6.5899 $\times \;10^{-7}$		

Lastly, we analyse the effect of factor D (speed of the obstacle) on this performance. The procedure that has been followed is shown in the tree graph of Figure A22, and the necessary ANOVA, in Table A5. Taking into account these results, the complete set of conclusions is:

- Factor D has no effect on the expected uncertainty of the filter given that B = 1.
- Factor D has no effect given that B = 2.

These conclusions allow us to assure that the speed of the obstacle has no relevant influence on the filter uncertainty, regardless of the value of the remaining factors. This behaviour can be observed in the histograms of Figure A23.

**Table A5:** One-way ANOVA table for factor D given B = 2.

Source	SS	df	MS	F	p-Value
D	1.5675 $\times \;10^{-9}$	1	1.5675 $\times \;10^{-9}$	0.0024	0.9611
Within cells	0.00319	4848	6.5899 $\times \;10^{-7}$		

### Appendix D.3. Convergence Performance

According to the established procedure, the first step is to perform a four-way ANOVA for the data corresponding to the performance of convergence of the filter. These results are shown in Table A6.

**Table A6:** Four-way ANOVA table for the convergence performance.

Source	SS	df	MS	F	p-Value
A	27.6910	1	27.6910	0.1702	0.6800
B	962,747.0726	1	962,747.0726	5917.3969	0.0000
C	1,601,490.7650	1	1,601,490.7650	9843.3501	0.0000
D	6.8851	1	6.8851	0.0423	0.8370
AxB	17.6499	1	17.6499	0.1085	0.7419
AxC	3.7469	1	3.7469	0.0230	0.8794
AxD	11.3538	1	11.3538	0.0698	0.7917
BxC	1,255,927.0726	1	1,255,927.0726	7719.3888	0.0000
BxD	672.7963	1	672.7963	4.1353	0.0421
CxD	61.5150	1	61.5150	0.3781	0.5387
AxBxC	78.7749	1	78.7749	0.4842	0.4866
AxBxD	35.0676	1	35.0676	0.2155	0.6425
AxCxD	17.1713	1	17.1713	0.1055	0.7453
BxCxD	0.0742	1	0.0742	0.0005	0.9830
AxBxCxD	20.9213	1	20.9213	0.1286	0.7199
Within cells	788,758.6	4848	162.7		

As before, we begin with the analysis of the effect of factor A (initial position of the obstacle) on the filter convergence. The procedure that has been followed is encoded in the tree graph of Figure A24 and the necessary ANOVA tables, in Figure A25. In this case, the complete set of conclusions is:

- Factor A has no effect on the convergence of the filter given that B = 1 and C = 1.
- Factor A has no effect given that B = 1 and C = 2.
- Factor A has no effect given that B = 2.

In order to complete this analysis correctly for the ANOVA assumptions, it has been necessary to force some interactions as indicated in Figure A24. With these results we can assure that the initial position of the obstacle has no influence on the convergence of the filter, regardless of the values of the remaining factors. Such behaviour can be observed in the histograms of Figure A26, where the difference in the mean is due to changes in the values of factors B and C, as we discuss later on.

Now we address the case of factor B (amount of missing observations). The corresponding analysis is encoded in the tree graph of Figure A27, and the ANOVA tables generated during the process are collected in Figure A28. These results lead to the following conclusions:

- Factor B has effect on the convergence of the filter given that C = 1.
- Factor B has no effect given that C = 2.

In this analysis we have used different omega squared measures. For interaction BxC (see Table A6), ${\hat{\omega }}^{2}=0.6134$, thus it is considered relevant. For the case of main effect B with C = 1, ${\hat{\omega }}^{2}=0.7362$, which is also considered very relevant. However, as opposed to the result of ANOVA table in Figure A28b, main effect B with C = 2 is not considered relevant enough, because it has ${\hat{\omega }}^{2}=0.0119$. With these results we can affirm that the amount of missing observations in the sensor has an important impact on the convergence of the filter only when the number of biased sensory observations is negligible. More specifically, an increase in the number of missing observations leads to a much slower convergence, as shown in the histograms of Figure A29a. This effect, however, nearly vanishes in the presence of biased observations (see histograms in Figure A29b). There, the increase of missing observations only leads to a higher variance, but only as a secondary effect.

We discuss now the effect of factor C (amount of biased observations) on the convergence performance. The resulting tree graph for this case is depicted in Figure A30 and the generated ANOVA tables during the process are shown in Figure A31. The complete set of conclusions for this situation is:

- Factor C has effect on the convergence of the filter given that B = 1.
- Factor C has no effect given that B = 2.

This analysis is very similar to the previous one. Omega squared values for both main effects of factor C indicated in the conclusions are ${\hat{\omega }}^{2}=0.7828$ and ${\hat{\omega }}^{2}=0.0129$ respectively, thus, the presence of biased observations in the sensor is relevant to the convergence of the filter only when there are no missing observations too. Also, in this case the increase of biased readings leads to a much slower convergence; the effect disappears when the presence of missing observations is high. These behaviours can be seen in the histograms of Figure A32a,b, respectively.

We finally proceed with the analysis of the effect of factor D (speed of the obstacle). The process being followed is encoded in the tree graph of Figure A33 and the used ANOVA tables are shown in Figure A34. In this case, the complete set of conclusions is:

- Factor D has no effect on the convergence of the filter given that B = 1 and C = 1.
- Factor D has no effect given that B = 1 and C = 2.
- Factor D has no effect given that B = 2.

All the interactions referenced in Figure A33 have been forced by us. Note also that we have considered irrelevant the BxD interaction (see Table A6) because its associated omega squared value is ${\hat{\omega }}^{2}=6.4417\times 10^{-4}$. The obtained conclusions allow us to affirm that the speed of the obstacle has no influence on the number of steps that lead to convergence in a filtering process, regardless of the value of the remaining factors. This can be seen in the histograms of Figure A35, where the changes in the population mean are only due to the effect of factors B and C, as we discussed before.
